# The Highly Controlled and Efficient Polymerization of Ethylene

**DOI:** 10.1002/anie.202216464

**Published:** 2023-01-13

**Authors:** Alexander Goller, Johannes Obenauf, Winfried P. Kretschmer, Rhett Kempe

**Affiliations:** ^1^ Anorganische Chemie II—Katalysatordesign Sustainable Chemistry Centre Universität Bayreuth Universitätsstraße 30, NW I 95440 Bayreuth Germany

**Keywords:** Catalyst Economy, Controlled Polymerization, Coordinative Chain Transfer Polymerization, Ethylene Polymerization, Zirconium

## Abstract

The highly controlled and efficient polymerization of ethylene is a very attractive but challenging target. Herein we report on a Coordinative Chain Transfer Polymerization catalyst, which combines a high degree of control and very high activity in ethylene oligo‐ or polymerization with extremely high chain transfer agent (triethylaluminum) to catalyst ratios (catalyst economy). Our Zr catalyst is long living and temperature stable. The chain length of the polyethylene products increases over time under constant ethylene feed or until a certain volume of ethylene is completely consumed to reach the expected molecular weight. Very high activities are observed if the catalyst elongates 60 000 or more alkyl chains and the polydispersity of the strictly linear polyethylene materials obtained are very low. The key for the combination of high control and efficiency seems to be a catalyst stabilized by only one strongly bound monoanionic N‐ligand.

## Graphical abstract

Coordinative chain transfer polymerization permits the controlled and efficient polymerization of ethylene, currently the most important monomer for plastic production. The key to the combination of control, very high activity, and an excellent catalyst economy seems to be a Group 4 metal complex stabilized by only one bulky and strongly bound monoanionic ligand. NH^+^B^−^=N,N’‐dimethylanilinium tetrakis (pentafluorophenyl)borate.

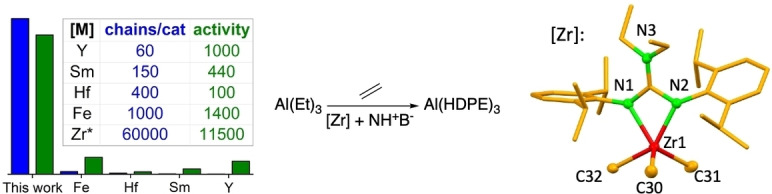

The controlled polymerization of ethylene, the most important monomer for plastic production, is a challenging and highly attractive target. Controlled polymerization protocols permit the synthesis of tailor‐made and functionalized polymers and are the basis for the formation of well‐defined multi‐block copolymers. The latter ones can undergo micro‐phase separation leading to an infinite variety of reproducibly formed polymer nanostructures.^[1]^ Living type of polymerization^[2]^ is one option (Figure 1A) for the controlled polymerization of ethylene and has been applied successfully to address challenges associated with a circular economy such as polyolefin plastic recycling.^[3]^ Unfortunately, living ethylene polymerization needs sophisticated coordination compounds as initiators and produces only one polymer chain per coordination compound. Coordinative Chain Transfer Polymerization (CCTP),^[4]^ a polymerization protocol in which inexpensive metal alkyls (Al, Mg, Zn for instance), resistant to ß‐H elimination in the used temperature range, are the initiators and the sophisticated coordination compound acts as polymerization catalyst (Figure 1B).^[5]^ A very fast chain transfer, fast relative to chain propagation, between the metal alkyls and the catalyst permits the insertion of only a few, preferentially one, ethylene molecules, while the alkyl or polymeryl chain is resting at the catalyst (reversible CCTP). The consequence is a homogeneous chain propagation permitting excellent control over the molecular weight of the obtained PE materials by time with a constant ethylene feed or by feeding a defined amount of ethylene (Figure 1B). In addition, each polymer chain is metal (Al, Mg or Zn) terminated. Unfortunately, CCTP is associated with a fundamental problem, that of chain efficiency or catalyst economy. The CTA binds to the active catalyst by blocking the site for monomer coordination in a rapidly maintained equilibrium forming heterobimetallic complexes,^[6]^ which are responsible for the chain transfer. Thus, the chain propagation rate or polymerization activity depends on the CTA concentration in an inverse first‐order.[^7^, ^13a^] This inverse first‐order dependence has restricted CCTP to very low CTA‐to‐catalyst ratios equate with a low number of polymer chains a catalyst molecule can grow in a controlled fashion (Figure 1C). Key catalyst development for reversible CCTP has been described by the groups of Mortreux,^[8]^ Gibson and Britovsek,^[9]^ us,^[10]^ Sita and co‐worker.^[11]^ Unfortunately, these CCTP catalysts can only extend a rather low number of alkyl chains (Figure 1C). The Mortreux lanthanoid catalyst (Sm in Figure 1C), mediating chain transfer to magnesium dialkyls, accepts only a CTA‐to‐catalyst ratio of 2 to reach an activity higher than 1000 kg mol^−1^ h^−1^ bar^−1^.^[8]^ Our Y catalyst (chain transfer to Al) is only slightly better, tolerating a CTA‐to‐catalyst ratio of 20 at similar activity,^[10]^ while early transition metal catalysts, for example the Sita Zr and Hf systems are using a diethyl zinc to catalyst ratio up to 200 but drop down in activity to 100 kg mol^−1^ h^−1^ bar^−1^.[^11a^, ^12^] Till now the most efficient CCTP catalyst, an iron compound (Fe in Figure 1 C), reaches a remarkable CTA‐to‐catalyst ratio of 550 (Et_2_Zn) at an activity of 1400 kg mol^−1^ h^−1^ bar^−1^ (Figure 1C).[^9b^, ^9c^] Further CCTP‐catalysts, which do not reach the 1000 kg mol^−1^ h^−1^ bar^−1^ activity with any reported CTA‐to‐catalyst ratio, are summarized in Table S2. CCTP has been used to synthesize well defined end‐group functionalized PE materials (reviews,^[13]^ starting from Zn‐termination,^[14]^ Mg‐termination,^[15]^ Al‐termination^[16]^) and block‐copolymers for microphase separation and material science.^[17]^ In addition, reversible CCTP has been combined with chain displacement catalysis (Ni catalyzed ß‐H elimination/transfer) permitting the spacial separation and independent tuning of chain propagation and chain termination.^[18]^ Recently, concepts for polydispersity control^[19]^ and the synthesis of highly melting polyolefin thermosplastic materials have been reported.^[20]^ These impressive polymer synthesis applications of CCTP do motivate the design of a catalyst (family) able to combine control, activity, and catalyst economy (in combination with an inexpensive CTA). By looking at the key equilibrium between the chain growing state and chain transfer state (Figure 1D, top), one could envision that the integration of both states into one molecule (Figure 1D, bottom) could increase catalyst economy significantly by permitting simultaneous chain transfer and chain propagation. Formally, a Group 4 metal catalyst carrying only one ancillary ligand to provide steric protection and catalyst solubility in nonpolar solvents could provide the necessary number of three additional anionic ligands to generate a cation (1) and host an aluminate (2) and the polymeryl chain (3) simultaneously (Figure 1D). Herein, we report on a Zr catalyst stabilized by one sterically demanding guanidinato ligand (Figure 2 right), which undergoes reversible CCTP with an inexpensive CTA, namely, triethylaluminum (TEAl). One catalyst molecule can mediate growth of 60 000 Al terminated polymeryl chains with activities of more than 11 000 kg mol^−1^ h^−1^ bar^−1^. This is an outstanding catalyst economy (Figure 1 C) combined with a very high activity. Our Zr catalyst is long‐term stable and temperature stable up to 70 °C at very high aluminum alkyl concentrations. The molecular weight of the Al‐terminated polyethylene (PE) products increases over time under constant ethylene feed and can be adjusted to reach the expected molecular weights from *M*_n_=86 g mol^−1^ (C6, shortest peak chain length) till about *M*_n_=2000 g mol^−1^. At about *M*_n_=2000 g mol^−1^, Al terminated PE starts to precipitates if CCTP is carried out at 70 °C. A catalytic extension of all aluminum alkyl chains is observed even at very high Al‐Zr ratios. Polydispersity of the strictly linear PE materials obtained after hydrolysis are between 1.1 and 1.3. The key for the combination of high control and efficiency seems to be a catalyst stabilized by only one bulky anionic N‐ligand and, thus, able to mediate chain propagation and chain transfer simultaneously.

**Figure 1:**
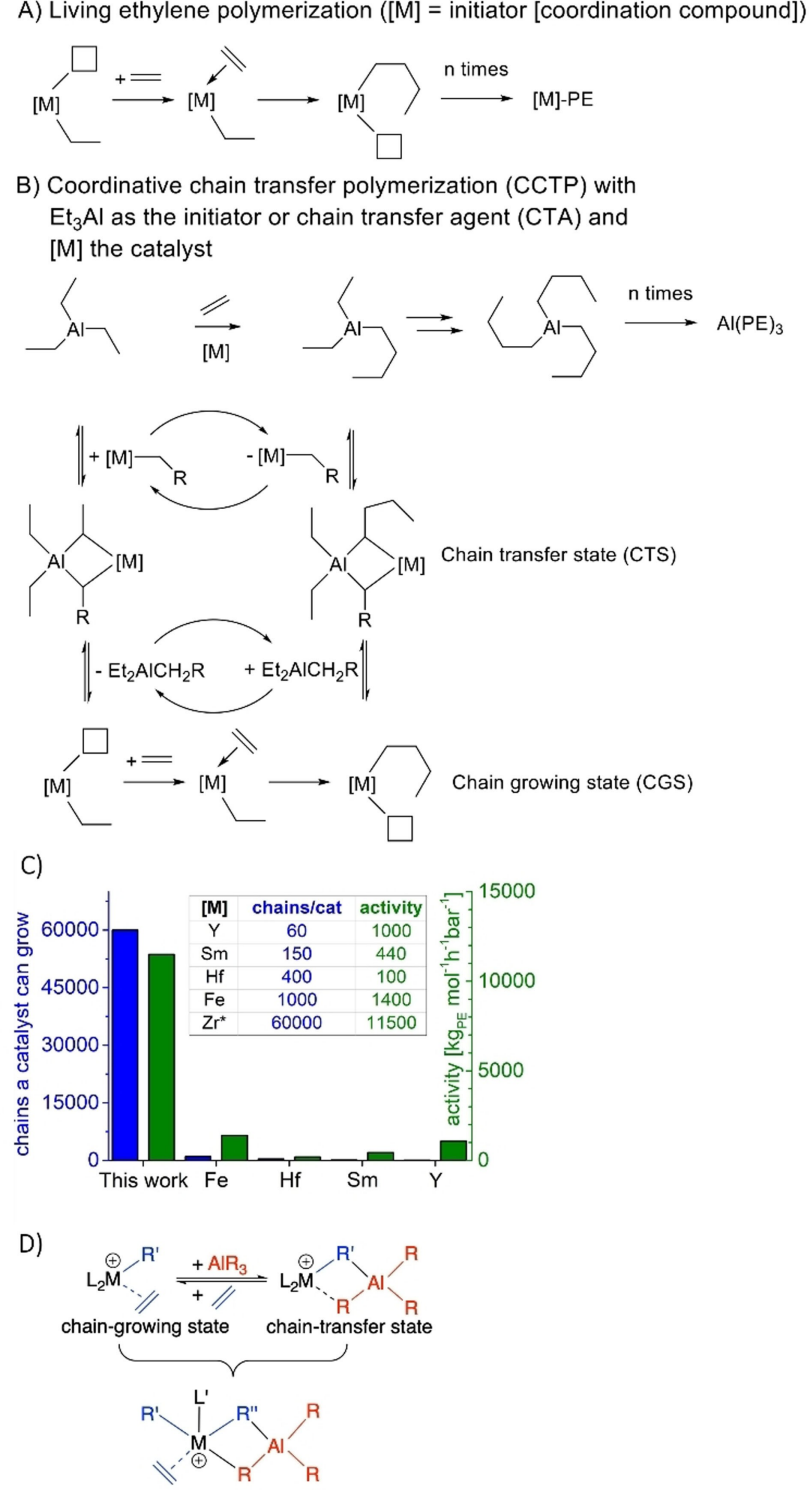
State of the art and catalyst reported here. A) Living ethylene polymerization. B) Coordinative Chain Transfer Polymerization (CCTP). C) Comparison of chains a catalyst can grow and activities for the most efficient reversible catalyst systems in the CCTP of ethylene Zr* (this work), Y,^[10]^ Sm,^[8a]^ Hf[^11a^, ^12^] and Fe^[9c]^ D) Key equilibrium in CCTP and catalyst design concept reported here. A Group 4 metal catalyst stabilized by one bulky anionic N‐ligand only is able to provide an active site for polymerization and chain transfer simultaneously (bifunctional catalyst). M=Group 4 metal and L=ancillary anionic ligands, L′ bulky guanidinato ligand.

**Figure 2:**
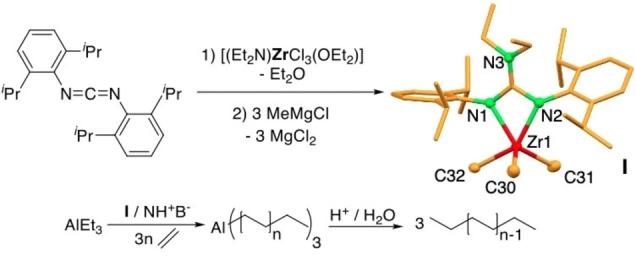
Synthesis and structure of the Zr complex **I** and reaction it catalyzes after activation with N,N′‐dimethylanilinium tetrakis(pentafluorophenyl)borate (NH^+^B^−^) (Et=ethyl).^[21]^

Precatalyst **I** was synthesized via the insertion of a sterically demanding carbodiimide into the Zr‐amido bond of the metal precursor [(Et_2_N)]ZrCl_3_(OEt_2_)]^[22]^ (Et=ethyl) generating the trichlorido intermediate. Precatalyst **I** can be obtained in good yields (74 %) as colorless crystals by the subsequent reaction with three equivalents of methylmagnesium chloride. The molecular structure of **I** was determined via single crystal X‐ray structure analysis and the molecular structure is shown in Figure 2. The Zr metal atom is stabilized by one bulky guanidinato‐ and three methyl ligands in a distorted trigonal bipyramidal manner with N2 and C32 in the apical positions. Instead of a 180° angle for a non‐distorted bipyramid, the C32−Zr1−N2 angle is scaled down by 37° to an angle of 142.7(1)°. The trigonal plane is formed by C30, C31 and N1. Selected bond lengths [Å] and angles [°]: Zr1−N1 2.238(3), Zr1−N2 2.201(2), Zr1−C30 2.240(3), Zr1−C31 2.228(4), Zr1−C32 2.260(3), C1−N1 1.338(4), C1−N2 1.366(4), C1−N3 1.346(4), N1−Zr1−N2 59.23(9) and N1−C1−N2 108.4(3).

Initial polymerization studies of **I**, activated with N,N′‐dimethylanilinium tetrakis(pentafluorophenyl)borate (NH^+^B^−^), in the presence of TEAl as a CTA showed very high polymerization activities in the presence of high amounts of CTA (Al‐to‐Zr ratio of 6000). A [NH^+^B^−^] : [Zr] ratio of 1.5 seemed to be optimal (Figure S7). The acidic workup of the trialkyl aluminum species yields strictly linear HDPE, as determined via nuclear magnetic resonance (NMR) spectroscopy. The absence of β‐H elimination/transfer reactions during CCTP can be confirmed via the absence of olefinic proton resonances in ^1^H NMR (Figure S20). A polymerization temperature of 50 °C resulted in the very good long‐term stability of the catalyst (Figure 3) with an activity of 5200 kg mol^−1^ h^−1^ bar^−1^. The measured ethylene flow was constant for 45 min after saturation and 10 minutes of the stabilization phase. A temperature increase leads to higher polymerization activities at 60 °C (15 900 kg mol^−1^ h^−1^ bar^−1^) and 70 °C (32 600 kg mol^−1^ h^−1^ bar^−1^) and initially also at 80 °C (Figure 3). A thermal decomposition of the active catalyst can be detected at 80 °C. An optimum of polymer solubility and polymerization activity was found at a temperature of 70 °C. The solubility of catalyst and chain elongated CTA under polymerization conditions is essential to maintain fast reversible chain transfer. The polymerization activity is independent from the Al : Zr ratio if the CTA concentration is kept constant (Figure 4). The ethylene uptake is almost equal in the shown experiments, demonstrating the high CTA tolerance also. The experiments are stopped after 3, 6, 9 and 12 liters of ethylene consumption to yield polymers with equal number average molecular weight of *M_n_* ≈480 g mol^−1^ and a very low dispersity of 1.13–1.03 after acidic work up. Next, we studied the CCTP process in the first few minutes of the reaction. For that, experiments with very high Al amounts (Al‐to‐Zr ratio of 50 000) and low ethylene conversions were carried out (Figure 5A). After hydrolysis of the aluminum alkyls, all samples were analyzed by GC. We found that the CCTP process start with a Schulz–Flory distribution of the product (α‐value=0.36) which slowly developed into higher propagation values.^[23]^ The maximum of the molecular weight distribution of the short chained products is shifted to higher values with increasing ethylene consumption, showing the highly reversible character of the CCTP process. (Figure 5 A; Figure S8–Figure S15; Table S6). A kinetic chain propagation one‐pot experiment with increasing amounts of ethylene was carried out next. Samples were taken after every 2 l of ethylene consumption, starting after 10 l (Figure 5 B and Table 1 entries 1–7). The increasing molecular weight with increasing ethylene conversion and low polymer dispersity *Ð* defined as *M*_w_/*M*_n_ indicates fast and reversible chain transfer between the catalyst and the CTA. Despite the high Al amount and Al‐to‐Zr ratio (14.6 mmol and 14 600 respectively), all aluminum alkyl chains become extended after the consumption of 12 l of ethylene, as indicated by N_exp_/N_theo_ ratios between 95 and 100 %.^[24]^ Consequently, the average molecular weight can be adjusted exactly varying the ethylene amount. In addition, *Ð* (1.2) remains constant over the entire polymerization process. Polymerization experiments with an Al‐to‐Zr ratio of 20 000 are listed in Table 2. Increasing amounts of ethylene lead to increasing polymer molecular weight and the full addressing of the CTA, while keeping *Ð* constant. The overall activities decrease, from 19 500 kg mol^−1^ h^−1^ bar^−1^ (14 l ethylene, entry 8) to 11 500 kg mol^−1^ h^−1^ bar^−1^ (22 l ethylene, entry 12) indicating a slow catalyst deactivation process or/and an activity reduction due to viscosity increase. NMR analysis of the products revealed the formation of strictly linear PE after hydrolysis of the aluminum alkyls. Furthermore, polymeryl alcohols (entry 13^[25]^) can be obtained by quenching the reaction with pure O_2_ confirming the presence of the Al‐termination. A hydroxyl functionalization of 69 mol % has been determined via ^1^H NMR. A different autoclave had to be used for the alcohol formation experiment, which explains the slightly different results between run 9 and 13 (Table 2). All alkyl chains of the CTA can be extended if the ethylene conversion is high enough (see Table 1+2).

**Figure 3:**
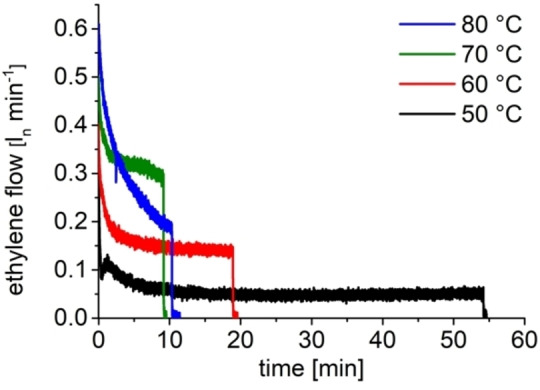
Determination of the temperature stability of the catalyst system based on **I** in the presence of 6000 equivalents of TEAl by monitoring of the ethylene flow. An amount of 3 l of ethylene were consumed per run. At 50 °C (black), ethylene consumption is constant for more than 45 min. At 80 °C, fast catalyst decomposition is observed suggesting lower temperatures as the optimum (70 °C, green). Higher polymerization temperatures are beneficial since higher molecular weight products can be formed in a highly controlled fashion due to better polymer solubility. Conditions: 1.5 bara ethylene, 3 l ethylene, 0.5 μmol **I**, 3 mmol TEAl, 0.75 μmol NH^+^B^−^, 75 ml toluene.

**Figure 4:**
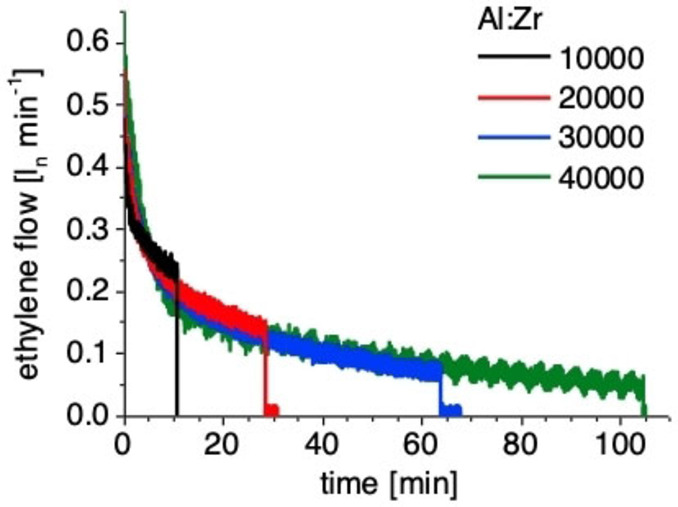
Ethylene flow plot of CCTP experiments with increasing Al : Zr ratios (Table S5). The TEAl‐concentration and catalyst amount was kept constant. The experiments were stopped after 3 (black), 6 (red), 9 (blue) and 12 (green) liters of ethylene consumption to synthesize polymers with equal average molecular weight (Mn is around 480 g mol^−1^). Conditions: 1.5 bara, 0.5 μmol **I**, c(TEAl) 66.6 μmol ml^−1^ in toluene; 75 ml (black); 150 ml (red); 225 ml (blue); 300 ml (green) toluene; 70 °C; 0.75 μmol NH^+^B^−^.

**Figure 5:**
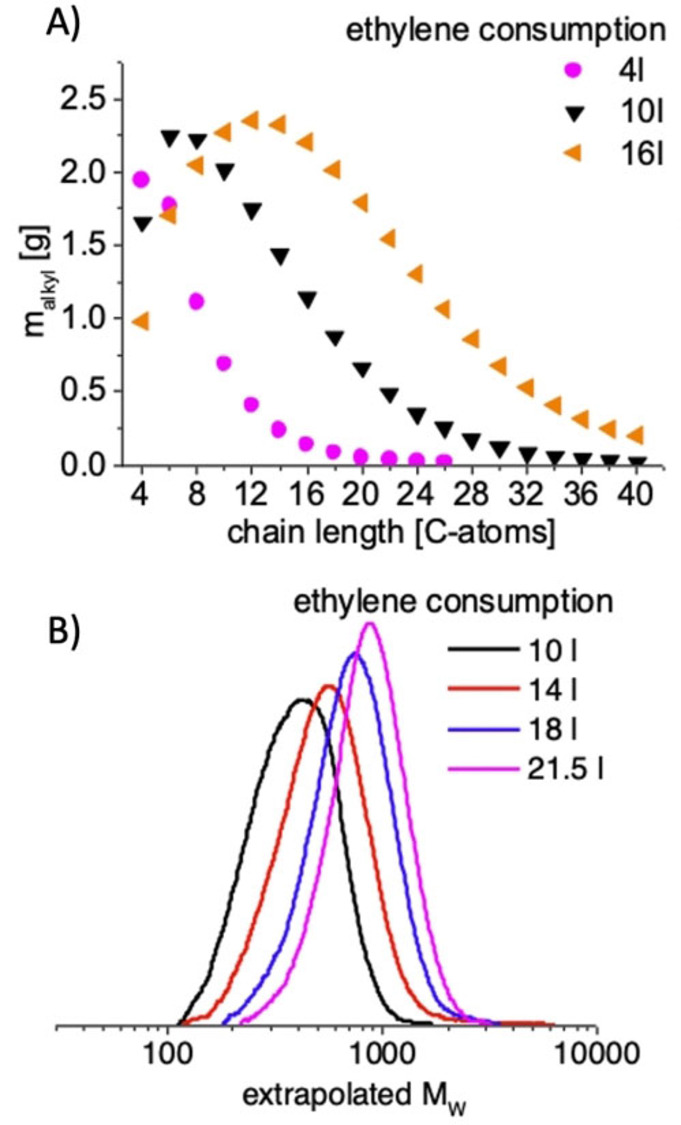
Chain propagation study by sequential sample taking. A) Polymerization experiments with high TEAl amount leading to short chain products. Only every second GC plot for clarity. Conditions: 1 μmol **I**; 2 bara ethylene, 250 mL toluene, 50 mmol TEAl, 1.1 μmol NH^+^B^−^. B) Experiments with lower TEAl amount to synthesize polymers (Table 1). The samples for the SEC measurements were either taken directly from the toluene solution (10 l) or from dried polymers (14 to 21.5 l). Only every second SEC plot is shown for clarity. Rising average molecular weight at increasing ethylene consumptions and low dispersity's (1.2) indicates a reversible CCTP reaction. Conditions: 1.5 bara ethylene, 1 μmol **I**, 14.6 mmol [Al], 1.5 μmol NH^+^B^−^, 275 ml toluene, sample taking every 2 l of ethylene consumption after 10 l of ethylene consumption, sample volume: 8 ml.

**Table 1:** Homopolymerization experiments of ethylene yielding Al‐terminated PE with adjustable molecular weights in continuous flow mode by sampling at certain ethylene consumption.

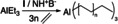				
entry	V_eth_	*Ð*	M_n_^[a]^	N_exp_/N_theo_
	[l]		[g mol^−1^]	[%]
1	10	1.2	350	89
2	12	1.2	400	95
3	14	1.2	470	96
4	16	1.2	550	96
5	18	1.2	650	95
6	20	1.2	720	97
7	21.5	1.2	780	99

1.5 bara ethylene, 1 μmol **I**, 14.6 mmol [Al], 1.5 μmol NH^+^B^−^, 275 ml toluene, sample taking every 2 l of ethylene consumption after 10 l of ethylene consumption, sample volume: 8 ml; [a] after acidic work up of the sample.

**Table 2:** Homopolymerization experiments of ethylene yielding Al‐terminated PE with adjustable molecular weights and elongation of all Al‐alkyl chains added.

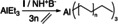					
entry	V_eth_	ethylene consumption^[b]^	*t*	M_n_^[c]^	N_exp_/N_theo_
	[l]	[kg mol^−1^ h^−1^ bar^−1^]	[min]	[g mol^−1^]	[%]
8	14	19 500	54	630	93
9	16	17 400	69	670	99
10	18	15 200	89	730	103
11	20	12 200	123	800	107
12	22	11 500	143	890	107
13^[a]^	16	14 800	82	760	93

2 bara ethylene; 0.5 μmol **I**; 10 mmol [Al]; 0.75 μmol NH^+^B^−^; 1000 rpm; 70 °C; 150 ml toluene, 300 ml steel autoclave; *Ð* of obtained polymer 1.3 in all experiments [a] quenched with pure O_2_ to yield polymeryl alcohols, 1000 ml steel autoclave; [b] Based on the time required until V_eth_ was consumed; [c] after acidic work up.

To explain the high CTA tolerance, we suggest a bifunctional polymerization catalyst enabling simultaneous chain propagation and chain transfer. To get an preliminary insight in the activation and active species of the Zr‐Al catalyst system based on **I**, ^1^H NMR experiments in C_6_D_5_Br were performed. Methane formation after the addition of NH^+^B^−^ suggests the elimination of one methyl group and the formation of a free coordination site. This site gets coordinated by the N,N′‐dimethylaniline formed (Figure 6 A). The aniline binding mode can be determined as a η^6^ coordination (aryl proton resonances 6.94 ppm; 6.34 ppm; 6.30 ppm; methyl proton resonance 2.73 ppm see Figure S24).[^26^, ^27^] Another ^1^H NMR experiment in CD_2_Cl_2_ was performed. By adding equimolar amounts of trimethylaluminum to **I**, three broad resonances at 0.33 ppm, −0.30 ppm and −0.78 ppm with corresponding integral values of 12, 6 and 6 protons can be detected (see Figure S34). These resonances could be assigned to four different species of methyl protons. Two CH_3_ groups of the guanidine backbone and two Zr−Me, which have the same resonance, two Zr−Me−Al and two Al−Me, respectively.[^6^, ^28^] This indicates a TMAl coordination modeling the chain transfer state (Figure 6 B). Interestingly, quantum chemically calculations have shown that tetramethylaluminate coordination to Zr is not favored in comparison to lanthanide ions.^[29]^ However, Zr has a strong tendency to reach an octahedral coordination sphere (see Figure S3), which is not achieved in the trimethyl complex **I**. The impact of rising TEAl concentrations on the polymerization activity was investigated, to see whether the activity of our Zr‐catalyst system still depends on the CTA concentration. A plot of the inverse Al concentrations against the activity indicates an inverse first‐order dependence of the activity from the Al concentration (Figure 6 C). In addition, very high polymerization activities were observed using 1/c(TEAl) of 50 l mol^−1^ (54 600 kg mol^−1^ h^−1^ bar^−1^). Changing the 1/c(TEAl) up to 10 l mol^−1^ led to a decrease in activity to 14 600 kg mol^−1^ h^−1^ bar^−1^, indicating that very high activities are observable even at such high amounts of CTA. The dependence of the activity on the Al concentration indicates that the active site of our catalyst ZrAl (Figure 6 D) is still coordinated by aluminum alkyls blocking the site for monomer coordination in a rapidly maintained equilibrium.^[7a]^ The very high activities still observed suggest that this coordination/blocking is very weak and the equilibrium between ZrAl and ZrAl_2_ under reaction conditions still permits the presence of significant amounts of ZrAl to catalyze the chain propagation very fast, even at very high Al concentrations. Both species, ZrAl and ZrAl_2_, can mediate chain transfer and ZrAl can mediate both chain propagation and transfer simultaneously (Figure 6D).

**Figure 6:**
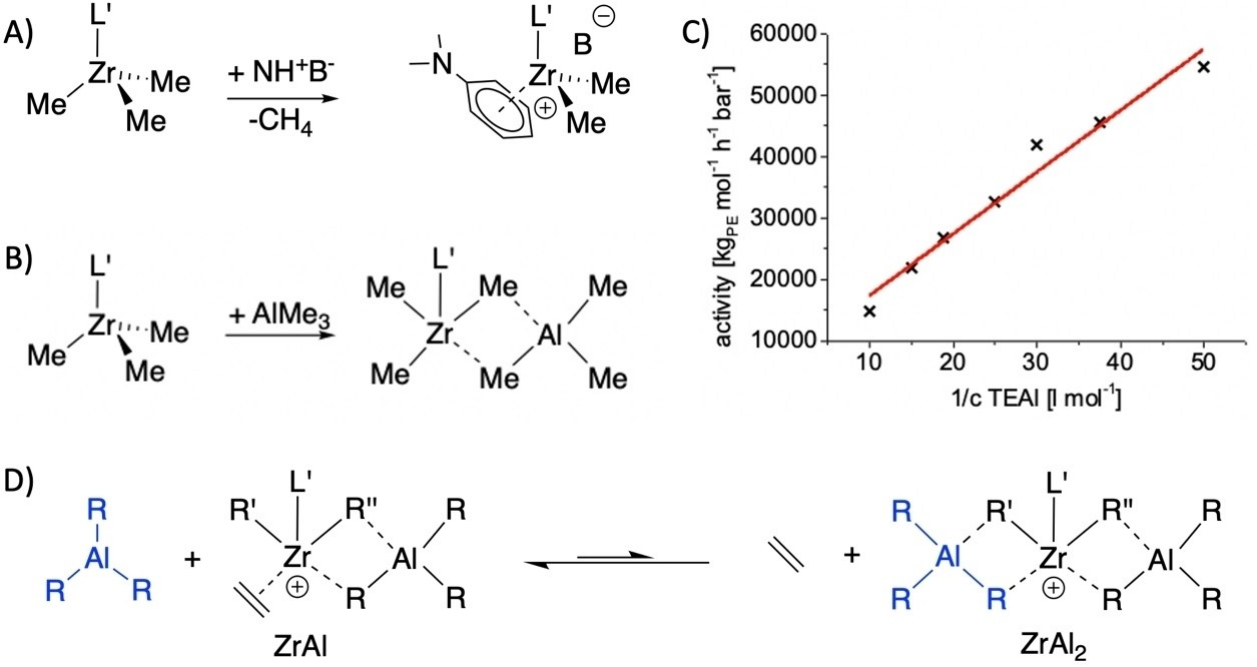
A) Activation of **I** with NH^+^B^−^ leads to an abstraction of one methyl group and the η^6^‐coordination of N,N′‐dimethylaniline. B) Equimolar addition of trimethylaluminum to **I** yields a bimetallic Zr−Al species. C) Plot of the dependency of the polymerization activity by the inverse Al concentration, indicating in combination with the high activities observed a weak coordination of a second aluminum alkyl under catalytic conditions. Conditions: 1.5 bara ethylene, 3 l ethylene, 0.5 μmol **I**, 0.75 μmol NH^+^B^−^, 75 ml toluene. D) Postulated equilibrium based on the activity dependency shown in C. For the Zr species shown on the left side of the equilibrium (ZrAl), simultaneous chain transfer and chain grows is proposed. Only chain transfer is observed for ZrAl_2_.

In conclusion, we report the highly controlled and efficient polymerization of ethylene. The key to the combination of control, high activity, and an extremely high number of polymeryl chains that get catalytically elongated (by one catalyst molecule) seems to be a bifunctional CCTP catalyst able to mediate very fast chain propagation and very fast chain transfer simultaneously. Group 4 metal complexes stabilized by one bulky monoanionic ligand only and the resulting bifunctional catalyst behavior might inspire CCTP catalyst development work for controlled olefin polymerizations.

## Conflict of interest

The authors declare the following competing financial interest(s): J. Obenauf, W. P. Kretschmer and R. Kempe are inventors of patent application US2018280951A1; W. P. Kretschmer and R. Kempe are inventors of patent application EP3294692A1. The remaining author declares no competing financial interests.

## Supporting information

As a service to our authors and readers, this journal provides supporting information supplied by the authors. Such materials are peer reviewed and may be re‐organized for online delivery, but are not copy‐edited or typeset. Technical support issues arising from supporting information (other than missing files) should be addressed to the authors.

Supporting Information

## Data Availability

The data that support the findings of this study are available in the supplementary material of this article.
