# Supplementary material for: The Highly Controlled and Efficient Polymerization of Ethylene
Source: Angew Chem Int Ed Engl. 2023 Jan 13;62(8):e202216464. doi: 10.1002/anie.202216464 (PMC10108248; doi:10.1002/anie.202216464)
Supplement: Supplementary file 1 — Supporting Information [file ANIE-62-0-s001.pdf]

Angewandte Chemie
Eine Zeitschrift der Gesellschaft Deutscher Chemiker

Supporting Information

The Highly Controlled and Efficient Polymerization of Ethylene

*A. Goller, J. Obenauf, W. P. Kretschmer, R. Kempe**

Supporting Information

This PDF file includes:

Materials and Methods
Instruments
Experimental Section
Tables S 1 to S 8
Figs. S 1 to S 35

Materials and Methods

General Methods

All manipulations of air sensitive compounds were performed with exclusion of oxygen and moisture using standard Schlenk techniques or a nitrogen or argon filled glove box (mBraun) with a high capacity circulator (< 0.1 ppm O_2). Deuterated solvents were purchased from Deutero GmbH. All solvents used for air and moisture sensitive reactions were dried and purified by distillation from Na/benzophenone or CaH_2 (halogenated solvents) under argon atmosphere.

Chemicals

$Zr(NEt_2)_4$[1] and $[(Et_2N)ZrCl_3(OEt_2)]$[2] were prepared according to the published procedures.

N,N-dimethylaniline (Sigma-Aldrich) was degassed and stirred over $LiAlH_4$ for 12 h and stored under argon prior to usage.

Triethyl aluminum ($AlEt_3$, TEAl, SASOL Germany GmbH), trimethyl aluminum ($AlMe_3$, TMAl, Sigma-Aldrich), cumene, methylmagnesium chloride (3.0 M in THF) (Sigma-Aldrich), dimethylanilinium tetrakis (pentafluorophenyl)borate (NH^+B^-), $ZrCl_4$ (ABCR), *N*,*N*'-bis(2,6-diisopropylphenyl) carbodiimide (TCI), *n*-buthyllithium (2.5 M in hexane) (Acros), ethanol p. a. (VWR), HCl (Kraft) were used without further purification. Toluene (Sigma-Aldrich, anhydrous, 99.8 %) and ethylene (Linde AG polymer grade) used for polymerization / oligomerization experiments were passed over columns of R3-11-supported Cu oxygen scavenger (BASF) and Al_2O_3 (Fischer Scientific). dMAO was obtained by removing the volatile components from a methylaluminoxane solution in toluene (Chemtura Europe Limited). Et_2NH (Acros) was degassed prior to usage.

Instruments

Elemental analysis (C,H,N):

Elemental analyses were carried out at a Vario Elementar EL III apparatus.

Gas chromatography (GC):

The toluene soluble fractions were analyzed at an Agilent 6890N gas chromatograph, equipped with a flame ionization detector and a (30 m x 0.32 mm x 0.25 µm) HP-5 column. To yield the correct amount of oligomer fractions the GC spectrum was corrected by response factors (Table S 1).

Table S 1. Response factors for the gas chromatograph in reference to cumene. The factors for C_6-C_{22} were determined for α-olefins, C_{24}-C_{36} for alkanes.

chain length	response factor	chain length	response factor
C_6	1.0143	C_{24}	0.9947
C_8	0.9789	C_{26}	1.0023
C_{10}	1.1839	C_{28}	0.9918
C_{12}	1.4259	C_{30}	0.9300
C_{14}	1.5207	C_{32}	0.7297
C_{16}	1.4788	C_{34}	0.6200
C_{18}	1.4559	C_{36}	0.4300
C_{20}	1.1864	C_{38}	0.3121
C_{22}	0.9615		

Gas chromatography – mass spectroscopy (GC-MS):

The GC-MS measurements were performed at an Agilent 7890A GC System equipped with a 5975 C inert MSD detector and a (30 m x 0.32 mm x 0.25 µm) HP-5 MS column.

Size-exclusion chromatography (SEC):

SEC analyses were carried out at an Agilent (Polymer Laboratories Ltd.) PL-SEC 220 high temperature chromatographic unit equipped with refractive index and differential pressure detectors, a guard and three linear mixed bed columns (Agilent Olexis). SEC analysis was performed at 150 °C using 1,2,4-trichlorobenzene as mobile phase. The samples were prepared by dissolving the polymer (0.1 wt.-%) in the mobile phase in an external oven and were run without filtration. The molecular weights of the samples were referenced to linear narrow HDPE standards (M_w = 110 - 430000 g mol^{-1}, K = 40.6 and α = 0.725).

Nuclear magnetic resonance spectroscopy (NMR):

The ^{1}H and ^{13}C spectra were performed at Varian INOVA 300 (^{1}H: 299.9 MHz, ^{13}C: 75.4 MHz), Varian INOVA 400 (^{1}H: 399.8 MHz, ^{19}F: 376.3 MHz ^{13}C: 100.5 MHz). The ^{1}H and ^{13}C NMR spectra were referenced internally to the residual solvent resonances. Chemical shifts (δ) are reported in ppm.

X-ray crystallography:

X-ray crystal structure analyses were performed at a STOE-IPDS II diffractometer [λ(Mo-$K\alpha$) = 0.71073 Å] equipped with an Oxford Cryostream low-temperature unit. Structure solution and refinement were accomplished with SIR97[3], SHELXL-*2013*[4] and WinGX.[5]

Polymerization autoclaves:

The typical polymerization experiments were carried out in a 300 ml stainless steel autoclave (BüchiGlasUster) with mechanical stirrer (1000 rpm). The temperature was controlled via an external water bath. The kinetic chain-growth and oxidation experiments were carried out in 1000 ml temperature and pressure controlled stainless steel autoclaves with bottom sample valve and mechanical stirrer (500 - 1000 rpm). The temperature control was ensured via a jacket combining electrical heating and water cooling. The monomer gas flow was monitored with a Bronkhorst High-Tech EI-Flow unit.

Table S 2. Catalyst systems for the highly reversible CCTP and their limits. The precatalysts are shown in **Fig. S 1**.

**precat**	**activator**	**CTA**	**activity**[a)]	**CTA/precataly**	**Mn**	**Đ**	**Source**
1	$B(C_6F_5)_3$	MAO	100	1000	oligomers	n.d.	[6]
2	-	TEAl	450	266	oligomers	1.2	[7]

a) $kg_{polymer}\ mol^{-1}\ h^{-1}\ bar^{-1}$

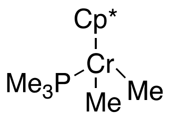

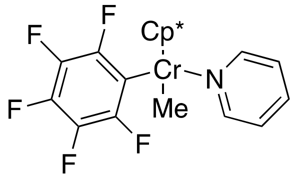

1 **2**

Fig. S 1. Precatalysts for the highly reversible CCTP. Further information can be seen in **Table S 2** (Cp*: η^5-pentamethylcyclopentadienido).

Experimental section

*Synthesis of di-µ-chlorido-bis[2,3-bis(2,6-diisopropylphenyl)-1,1-diethylguanidinato-tetrachlorido-zirconium(IV)] (**0**):*

$[(Et_2N)ZrCl_3(OEt_2)]$ (6.736 g, 19.6 mmol) and *N*,*N'*-bis(2,6-diisopropylphenyl) carbodiimide (7.103 g, 19.6 mmol) were dissolved in toluene (100 ml) and stirred for 24 hours at 60 °C. Toluene was removed under reduced pressure and the residue washed with hexane (30 ml). Drying in vacuum gave a colorless solid. Crystals suitable for X-ray single crystal analysis were obtained by layering a toluene solution with hexane. Yield 12.6 g (91 %). Crystallographic data and the molecular structure can be seen in Table S 3 and Fig. S 3.

^{1}H NMR (300 MHz, C_6D_6): δ = 7.04–7.13 [s, 6 H, Ar*H*]; 3.55 [m, 4 H, C*H*$(CH_3)_2$]; 2.61 [q, ^{3}J = 6.8 Hz, 4 H, N(C*H*$_2$CH$_3$)$_2$]; 1.50 [d, ^{3}J = 6.6 Hz, 12 H, CH(C*H*$_3$)$_2$]; 1.18 [d, ^{3}J = 6.8Hz, 12 H, CH(C*H*$_3$)$_2$]; 0.23 [t, ^{3}J = 7.0 Hz, 6 H, N(CH$_2$C*H*$_3$)$_2$] ppm. ^{13}C NMR (75.4 MHz, C_6D_6): δ = 169.9 (N*C*N); 143.5 (Ar*C*); 141.0; 127.3; 124.8; 110.3; 41.5 [N(*C*H$_2$CH$_3$)$_2$]; 29.0 [*C*H(CH$_3$)$_2$]; 26.1 [CH(*C*H$_3$)$_2$]; 24.2 [CH(*C*H$_3$)$_2$]; 11.4 [N(CH$_2$*C*H$_3$)$_2$] ppm. Elemental analysis: $C_{58}H_{88}Cl_6N_6Zr_2$ (1264.53) calcd. C 55.09, H 7.01, N 6.65; found C 54.13 H 6.95, N 6.53.

*Synthesis of 2,3-bis(2,6-diisopropylphenyl)-1,1-diethylguanidinato trimethanido zirconium(IV) (**I**):*

To a suspension of **0** (1176 mg, 0.93 mmol) in hexane (50 ml) methyl magnesium chloride solution in THF (1.96 ml, 5.88 mmol, 3 M) was added dropwise at -78 °C. The mixture was warmed to room temperature and stirred overnight. Storage of the concentrated filtrate at -30 °C led to colorless crystals. Yield 903 mg (85 %). Crystallographic data and the molecular structure can be seen in Table S 3 and Fig. S 4.

^{1}H NMR (300 MHz, C_6D_6): δ = 7.09 (s, 6 H, Ar*H*); 3.62 [sept, ^{3}J = 6.8 Hz, 4 H, C*H*(CH$_3$)$_2$]; 2.74 [q, ^{3}J = 7.1 Hz, 4 H, N(C*H*$_2$CH$_3$)$_2$]; 1.36 [d, ^{3}J = 7.1 Hz, 12 H, CH(C*H*$_3$)$_2$]; 1.24 [d, ^{3}J = 6.9Hz, 12 H, CH(C*H*$_3$)$_2$]; 0.86 [s, 9 H, Zr(C*H*$_3$)$_3$]; 0.26 [t, ^{3}J = 7.1 Hz, 6 H, N(CH$_2$C*H*$_3$)$_2$]; ppm. ^{13}C NMR (75.4 MHz, C_6D_6): δ = 169.5 (N*C*N); 143.3 (Ar*C*); 142.7; 125.5; 124.3;

51.4 [Zr(*C*H3)3]; 40.8 [N(*C*H2CH3)2]; 28.5 [*C*H(CH3)2]; 25.9 [CH(*C*H3)2]; 24.0 [CH(*C*H3)2]; 11.3 [N(CH2*C*H3)2] ppm. Elemental analysis: $C_{32}H_{53}N_3Zr$ (571.01): calcd. C 67.31, H 9.36, N 7.36; found C 67.15, H 9.46, N 7.33.

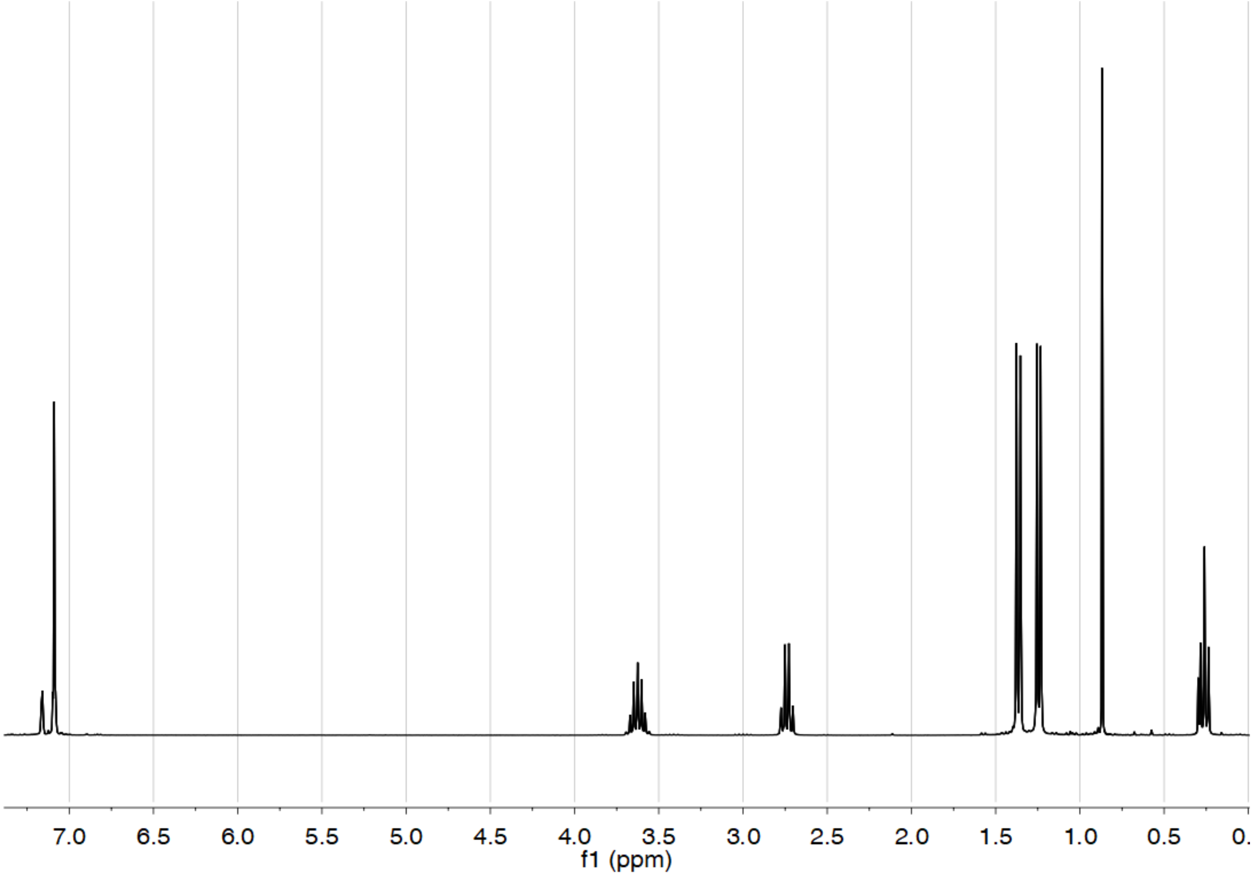

Fig. S 2. 300 MHz ^{1}H NMR spectra of **I** in C_6D_6.

Table S 3. Crystallographic data of the compounds characterized by single crystal X-ray structure analysis.

**compound**	**I**	**0**
Formula	$C_{32}H_{53}N_3Zr$	$C_{58}H_{88}Cl_6N_6Zr_2$, 2 (C_7H_8)
Formula weight	570.99	1448.75
Crystal system	monoclinic	monoclinic
Space group	$P2_1/c$	$P2_1/n$
a [Å]	9.4553(4)	10.9383(10)

b [Å]	17.2321(7)	20.6555(18)
c [Å]	10.3607(4)	16.4615(16)
α [°]	90	90
β [°]	108.682(3)	100.105(5)
γ [°]	90	90
Cell volume [$Å^3$]	1599.17(11)	3661.6(6)
Z	2	2
Crystal size [mm^3]	0.177x0.159x0.147	0.267x0.190x0.163
Habit	block	block
Color	Colorless	Colorless
Density [gcm^{-3}]	1.186	1.414
T [K]	133(2)	133
Theta range	2.08–27.11	1.26–25.65
Unique reflections	6693	6909
Observed reflections [I > 2s(I)]	5468	3487
Parameters	338	399
wR_2 (all data)	0.0724	0.2158
R [I > 2s(I)]	0.0379	0.0872
X-ray source	*λ*(Mo-*K*α) = 0.71073 Å	*λ*(Mo-*K*α) = 0.71073 Å
Deposition number	2048349	2048343

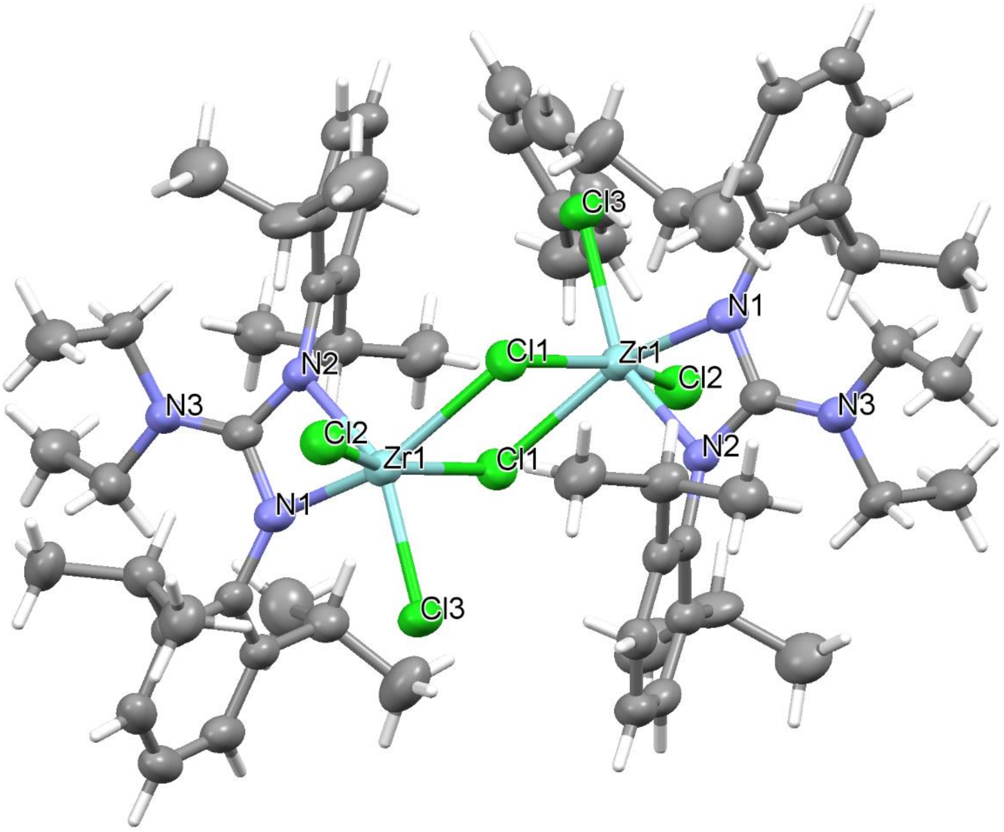

Fig. S 3. Molecular structure of compound **0** investigated by single crystal structure analysis. The atoms are displayed as probability ellipsoid. The hydrogen atoms are shown as capped sticks for clarity reasons

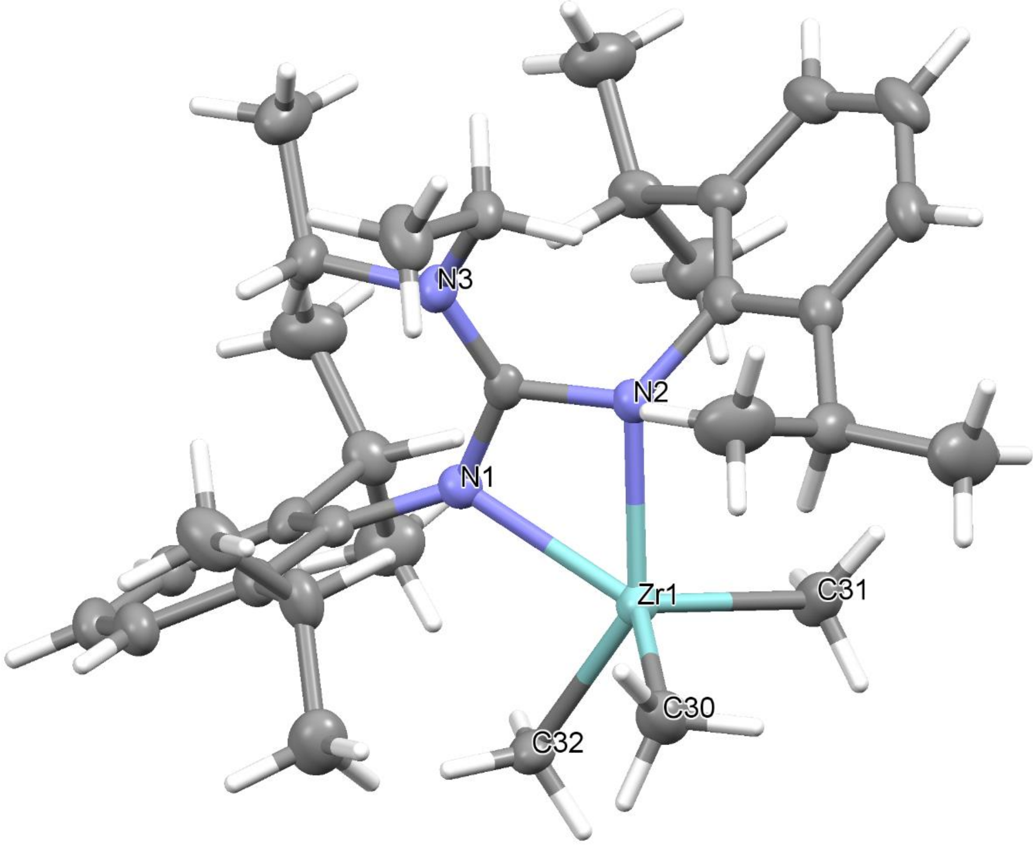

Fig. S 4. Molecular structure of compound **I** investigated by single crystal structure analysis. The atoms are displayed as probability ellipsoid. The hydrogen atoms are shown as capped sticks for clarity reasons.

CIF File compound **0:**

data_j75mp-neu

_audit_creation_method SHELXL-97

_chemical_name_systematic

;

 ?

;

_chemical_name_common ?

_chemical_melting_point ?

_chemical_formula_moiety 'C36 H52 Cl3 N3 Zr'

_chemical_formula_sum
 'C36 H52 Cl3 N3 Zr'
_chemical_formula_weight   724.38

loop_
 _atom_type_symbol
 _atom_type_description
 _atom_type_scat_dispersion_real
 _atom_type_scat_dispersion_imag
 _atom_type_scat_source
 'C' 'C'   0.0033   0.0016
 'International Tables Vol C Tables 4.2.6.8 and 6.1.1.4'
 'H' 'H'   0.0000   0.0000
 'International Tables Vol C Tables 4.2.6.8 and 6.1.1.4'
 'N' 'N'   0.0061   0.0033
 'International Tables Vol C Tables 4.2.6.8 and 6.1.1.4'
 'Cl' 'Cl'   0.1484   0.1585
 'International Tables Vol C Tables 4.2.6.8 and 6.1.1.4'
 'Zr' 'Zr' -2.9673   0.5597
 'International Tables Vol C Tables 4.2.6.8 and 6.1.1.4'

_symmetry_cell_setting   monoclinic
_symmetry_space_group_name_H-M   P21/n

loop_
 _symmetry_equiv_pos_as_xyz
 'x, y, z'
 '-x+1/2, y+1/2, -z+1/2'
 '-x, -y, -z'
 'x-1/2, -y-1/2, z-1/2'

```
_cell_length_a                    10.9383(10)
_cell_length_b                    20.6555(18)
_cell_length_c                    16.4615(16)
_cell_angle_alpha                  90.00
_cell_angle_beta                  100.105(5)
_cell_angle_gamma                  90.00
_cell_volume                      3661.6(6)
_cell_formula_units_Z              4
_cell_measurement_temperature     133(2)
_cell_measurement_reflns_used     15189
_cell_measurement_theta_min       1.26
_cell_measurement_theta_max       25.65

_exptl_crystal_description        block
_exptl_crystal_colour             colourless
_exptl_crystal_size_max           0.267
_exptl_crystal_size_mid           0.190
_exptl_crystal_size_min           .0163
_exptl_crystal_density_meas       ?
_exptl_crystal_density_diffrn     1.314
_exptl_crystal_density_method     'not measured'
_exptl_crystal_F_000              1520
_exptl_absorpt_coefficient_mu     0.547
_exptl_absorpt_correction_type    numerical
_exptl_absorpt_correction_T_min   0.8532
_exptl_absorpt_correction_T_max   0.9489
_exptl_absorpt_process_details    ?

_exptl_special_details
;
 ?
```

;

```
_diffrn_ambient_temperature       133(2)
_diffrn_radiation_wavelength      0.71069
_diffrn_radiation_type            MoK\a
_diffrn_radiation_source          'fine-focus sealed tube'
_diffrn_radiation_monochromator   graphite
_diffrn_measurement_device_type   STOE-IPDS-II
_diffrn_measurement_method        omega-scan
_diffrn_detector_area_resol_mean  ?
_diffrn_standards_number          ?
_diffrn_standards_interval_count  ?
_diffrn_standards_interval_time   ?
_diffrn_standards_decay_%         ?
_diffrn_reflns_number             47477
_diffrn_reflns_av_R_equivalents   0.2472
_diffrn_reflns_av_sigmaI/netI     0.1479
_diffrn_reflns_limit_h_min        -13
_diffrn_reflns_limit_h_max        13
_diffrn_reflns_limit_k_min        -25
_diffrn_reflns_limit_k_max        25
_diffrn_reflns_limit_l_min        -20
_diffrn_reflns_limit_l_max        20
_diffrn_reflns_theta_min          1.60
_diffrn_reflns_theta_max          25.69
_reflns_number_total              6909
_reflns_number_gt                 3487
_reflns_threshold_expression      >2\s(I)

_computing_data_collection        STOE-XAREA
_computing_cell_refinement        STOE-XAREA
```

_computing_data_reduction STOE-XAREA
_computing_structure_solution Sir-97
_computing_structure_refinement 'SHELXL-97 (Sheldrick, 2008)'
_computing_molecular_graphics ?
_computing_publication_material ?

_refine_special_details
;
Refinement of F^2^ against ALL reflections. The weighted R-factor wR and goodness of fit S are based on F^2^, conventional R-factors R are based on F, with F set to zero for negative F^2^. The threshold expression of F^2^ > 2\s(F^2^) is used only for calculating R-factors(gt) etc. and is not relevant to the choice of reflections for refinement. R-factors based on F^2^ are statistically about twice as large as those based on F, and R-factors based on ALL data will be even larger.
;

_refine_ls_structure_factor_coef Fsqd
_refine_ls_matrix_type full
_refine_ls_weighting_scheme calc
_refine_ls_weighting_details
'calc w=1/[\s^2^(Fo^2^)+(0.1075P)^2^+0.0000P] where P=(Fo^2^+2Fc^2^)/3'
_atom_sites_solution_primary direct
_atom_sites_solution_secondary difmap
_atom_sites_solution_hydrogens geom
_refine_ls_hydrogen_treatment constr
_refine_ls_extinction_method none
_refine_ls_extinction_coef ?
_refine_ls_number_reflns 6909
_refine_ls_number_parameters 399
_refine_ls_number_restraints 0

_refine_ls_R_factor_all 0.1543

_refine_ls_R_factor_gt 0.0872

_refine_ls_wR_factor_ref 0.2158

_refine_ls_wR_factor_gt 0.1900

_refine_ls_goodness_of_fit_ref 0.914

_refine_ls_restrained_S_all 0.914

_refine_ls_shift/su_max 0.001

_refine_ls_shift/su_mean 0.000

loop_

_atom_site_label

_atom_site_type_symbol

_atom_site_fract_x

_atom_site_fract_y

_atom_site_fract_z

_atom_site_U_iso_or_equiv

_atom_site_adp_type

_atom_site_occupancy

_atom_site_symmetry_multiplicity

_atom_site_calc_flag

_atom_site_refinement_flags

_atom_site_disorder_assembly

_atom_site_disorder_group

Zr1 Zr -0.07158(6) 0.08771(4) 0.02604(4) 0.0424(2) Uani 1 1 d . . .

Cl1 Cl 0.12023(17) 0.03539(9) -0.01936(12) 0.0490(5) Uani 1 1 d . . .

Cl2 Cl -0.25985(17) 0.09608(10) 0.08003(12) 0.0536(5) Uani 1 1 d . . .

Cl3 Cl 0.06136(18) 0.11797(10) 0.15171(12) 0.0538(5) Uani 1 1 d . . .

N1 N -0.0410(5) 0.1829(3) -0.0196(4) 0.0448(15) Uani 1 1 d . . .

N2 N -0.1512(5) 0.1126(3) -0.1001(4) 0.0401(14) Uani 1 1 d . . .

C1 C -0.1155(6) 0.1751(4) -0.0955(4) 0.0398(17) Uani 1 1 d . . .

N3 N -0.1532(5) 0.2212(3) -0.1507(4) 0.0445(15) Uani 1 1 d . . .

C2 C -0.2730(6) 0.2136(4) -0.2076(5) 0.0486(19) Uani 1 1 d . . .
H2A H -0.2956 0.1672 -0.2120 0.058 Uiso 1 1 calc R . .
H2B H -0.2642 0.2291 -0.2631 0.058 Uiso 1 1 calc R . .
C3 C -0.3755(8) 0.2514(5) -0.1775(6) 0.072(3) Uani 1 1 d . . .
H3A H -0.3511 0.2969 -0.1701 0.108 Uiso 1 1 calc R . .
H3B H -0.3894 0.2335 -0.1247 0.108 Uiso 1 1 calc R . .
H3C H -0.4522 0.2481 -0.2182 0.108 Uiso 1 1 calc R . .
C4 C -0.0975(8) 0.2867(4) -0.1457(6) 0.059(2) Uani 1 1 d . . .
H4A H -0.0104 0.2837 -0.1167 0.071 Uiso 1 1 calc R . .
H4B H -0.1430 0.3146 -0.1122 0.071 Uiso 1 1 calc R . .
C5 C -0.0994(9) 0.3181(4) -0.2277(5) 0.066(2) Uani 1 1 d . . .
H5A H -0.0619 0.2889 -0.2634 0.099 Uiso 1 1 calc R . .
H5B H -0.0521 0.3586 -0.2202 0.099 Uiso 1 1 calc R . .
H5C H -0.1854 0.3273 -0.2534 0.099 Uiso 1 1 calc R . .
C6 C 0.0113(7) 0.2417(4) 0.0187(4) 0.0455(18) Uani 1 1 d . . .
C7 C -0.0599(7) 0.2831(4) 0.0593(5) 0.050(2) Uani 1 1 d . . .
C8 C -0.0025(8) 0.3383(4) 0.0985(5) 0.052(2) Uani 1 1 d . . .
H8 H -0.0485 0.3665 0.1274 0.062 Uiso 1 1 calc R . .
C9 C 0.1194(8) 0.3521(4) 0.0955(5) 0.059(2) Uani 1 1 d . . .
H9 H 0.1563 0.3906 0.1202 0.070 Uiso 1 1 calc R . .
C10 C 0.1882(8) 0.3101(4) 0.0568(5) 0.053(2) Uani 1 1 d . . .
H10 H 0.2734 0.3193 0.0572 0.064 Uiso 1 1 calc R . .
C11 C 0.1371(7) 0.2551(4) 0.0176(5) 0.0451(18) Uani 1 1 d . . .
C12 C 0.2175(7) 0.2114(4) -0.0245(5) 0.053(2) Uani 1 1 d . . .
H12 H 0.1663 0.1725 -0.0443 0.064 Uiso 1 1 calc R . .
C13 C 0.3315(8) 0.1871(5) 0.0341(6) 0.077(3) Uani 1 1 d . . .
H13A H 0.3856 0.2238 0.0536 0.115 Uiso 1 1 calc R . .
H13B H 0.3768 0.1563 0.0051 0.115 Uiso 1 1 calc R . .
H13C H 0.3055 0.1656 0.0813 0.115 Uiso 1 1 calc R . .
C14 C 0.2561(9) 0.2428(5) -0.1008(6) 0.074(3) Uani 1 1 d . . .
H14A H 0.1818 0.2567 -0.1391 0.112 Uiso 1 1 calc R . .

H14B H 0.3020 0.2112 -0.1283 0.112 Uiso 1 1 calc R . .

H14C H 0.3091 0.2803 -0.0837 0.112 Uiso 1 1 calc R . .

C15 C -0.1959(7) 0.2699(4) 0.0663(5) 0.052(2) Uani 1 1 d . . .

H15 H -0.2246 0.2319 0.0303 0.063 Uiso 1 1 calc R . .

C16 C -0.2811(8) 0.3264(4) 0.0368(6) 0.067(2) Uani 1 1 d . . .

H16A H -0.2634 0.3623 0.0759 0.100 Uiso 1 1 calc R . .

H16B H -0.3677 0.3129 0.0331 0.100 Uiso 1 1 calc R . .

H16C H -0.2670 0.3403 -0.0177 0.100 Uiso 1 1 calc R . .

C17 C -0.2037(8) 0.2511(5) 0.1549(5) 0.060(2) Uani 1 1 d . . .

H17A H -0.1458 0.2155 0.1726 0.090 Uiso 1 1 calc R . .

H17B H -0.2885 0.2372 0.1578 0.090 Uiso 1 1 calc R . .

H17C H -0.1818 0.2885 0.1912 0.090 Uiso 1 1 calc R . .

C18 C -0.1825(6) 0.0737(3) -0.1747(4) 0.0394(17) Uani 1 1 d . . .

C19 C -0.1000(6) 0.0666(3) -0.2301(4) 0.0422(18) Uani 1 1 d . . .

C20 C -0.1311(7) 0.0238(4) -0.2952(5) 0.0455(18) Uani 1 1 d . . .

H20 H -0.0752 0.0183 -0.3328 0.055 Uiso 1 1 calc R . .

C21 C -0.2398(7) -0.0109(4) -0.3074(5) 0.050(2) Uani 1 1 d . . .

H21 H -0.2580 -0.0402 -0.3523 0.059 Uiso 1 1 calc R . .

C22 C -0.3217(7) -0.0028(4) -0.2542(5) 0.0473(19) Uani 1 1 d . . .

H22 H -0.3973 -0.0265 -0.2635 0.057 Uiso 1 1 calc R . .

C23 C -0.2982(7) 0.0387(4) -0.1877(5) 0.0466(19) Uani 1 1 d . . .

C24 C -0.3947(7) 0.0468(5) -0.1328(5) 0.059(2) Uani 1 1 d . . .

H24 H -0.3587 0.0746 -0.0847 0.071 Uiso 1 1 calc R . .

C25 C -0.4345(9) -0.0168(6) -0.1004(6) 0.083(3) Uani 1 1 d . . .

H25A H -0.4831 -0.0414 -0.1459 0.124 Uiso 1 1 calc R . .

H25B H -0.4854 -0.0085 -0.0581 0.124 Uiso 1 1 calc R . .

H25C H -0.3609 -0.0417 -0.0763 0.124 Uiso 1 1 calc R . .

C26 C -0.5105(8) 0.0822(5) -0.1827(7) 0.075(3) Uani 1 1 d . . .

H26A H -0.4834 0.1211 -0.2087 0.113 Uiso 1 1 calc R . .

H26B H -0.5670 0.0946 -0.1453 0.113 Uiso 1 1 calc R . .

H26C H -0.5536 0.0531 -0.2254 0.113 Uiso 1 1 calc R . .

C27 C 0.0201(7) 0.1052(4) -0.2243(5) 0.053(2) Uani 1 1 d . . .
H27 H 0.0362 0.1277 -0.1697 0.063 Uiso 1 1 calc R . .
C28 C 0.0035(8) 0.1566(4) -0.2920(6) 0.061(2) Uani 1 1 d . . .
H28A H -0.0005 0.1356 -0.3458 0.091 Uiso 1 1 calc R . .
H28B H 0.0740 0.1866 -0.2825 0.091 Uiso 1 1 calc R . .
H28C H -0.0736 0.1805 -0.2912 0.091 Uiso 1 1 calc R . .
C29 C 0.1334(7) 0.0627(5) -0.2310(6) 0.063(2) Uani 1 1 d . . .
H29A H 0.1437 0.0298 -0.1874 0.095 Uiso 1 1 calc R . .
H29B H 0.2080 0.0898 -0.2249 0.095 Uiso 1 1 calc R . .
H29C H 0.1206 0.0414 -0.2850 0.095 Uiso 1 1 calc R . .
C30 C -0.1615(11) 0.0974(5) 0.3130(6) 0.073(3) Uani 1 1 d . . .
H30 H -0.1583 0.1132 0.2592 0.088 Uiso 1 1 calc R . .
C31 C -0.2707(9) 0.0750(4) 0.3299(5) 0.060(2) Uani 1 1 d . . .
H31 H -0.3421 0.0752 0.2877 0.072 Uiso 1 1 calc R . .
C32 C -0.2806(7) 0.0515(4) 0.4078(5) 0.056(2) Uani 1 1 d . . .
C33 C -0.1710(8) 0.0525(4) 0.4680(5) 0.058(2) Uani 1 1 d . . .
H33 H -0.1731 0.0368 0.5220 0.070 Uiso 1 1 calc R . .
C34 C -0.0637(8) 0.0753(4) 0.4496(6) 0.063(2) Uani 1 1 d . . .
H34 H 0.0082 0.0758 0.4915 0.075 Uiso 1 1 calc R . .
C35 C -0.0546(9) 0.0980(5) 0.3722(7) 0.070(3) Uani 1 1 d . . .
H35 H 0.0219 0.1135 0.3599 0.085 Uiso 1 1 calc R . .
C36 C -0.3998(9) 0.0281(6) 0.4284(7) 0.082(3) Uani 1 1 d . . .
H36A H -0.4489 0.0652 0.4413 0.123 Uiso 1 1 calc R . .
H36B H -0.3830 -0.0008 0.4762 0.123 Uiso 1 1 calc R . .
H36C H -0.4461 0.0046 0.3811 0.123 Uiso 1 1 calc R . .

loop_
_atom_site_aniso_label
_atom_site_aniso_U_11
_atom_site_aniso_U_22
_atom_site_aniso_U_33

_atom_site_aniso_U_23
_atom_site_aniso_U_13
_atom_site_aniso_U_12
Zr1 0.0414(4) 0.0402(4) 0.0416(4) 0.0002(4) -0.0036(3) -0.0005(3)
Cl1 0.0444(10) 0.0409(11) 0.0589(12) -0.0008(9) 0.0009(9) -0.0028(8)
Cl2 0.0497(10) 0.0565(13) 0.0540(11) -0.0032(10) 0.0075(9) -0.0017(9)
Cl3 0.0561(11) 0.0511(12) 0.0466(11) -0.0018(9) -0.0116(9) -0.0033(9)
N1 0.043(3) 0.042(4) 0.044(4) 0.002(3) -0.007(3) -0.007(3)
N2 0.042(3) 0.035(3) 0.038(3) 0.001(3) -0.007(3) -0.001(3)
C1 0.034(4) 0.042(5) 0.041(4) -0.004(3) 0.000(3) -0.002(3)
N3 0.045(3) 0.039(4) 0.044(3) -0.001(3) -0.007(3) -0.001(3)
C2 0.045(4) 0.053(5) 0.041(4) 0.001(4) -0.011(3) 0.000(4)
C3 0.050(5) 0.073(6) 0.084(7) -0.021(5) -0.014(5) 0.004(4)
C4 0.063(5) 0.042(5) 0.064(5) 0.001(4) -0.011(4) 0.000(4)
C5 0.080(6) 0.051(5) 0.060(6) 0.008(4) -0.009(5) -0.012(4)
C6 0.055(5) 0.040(4) 0.036(4) -0.001(3) -0.010(3) 0.003(4)
C7 0.054(5) 0.042(5) 0.047(4) 0.002(4) -0.010(4) -0.004(4)
C8 0.071(5) 0.042(5) 0.038(4) -0.002(3) -0.004(4) 0.007(4)
C9 0.076(6) 0.040(5) 0.051(5) 0.000(4) -0.014(4) -0.008(4)
C10 0.061(5) 0.047(5) 0.045(5) 0.004(4) -0.012(4) -0.008(4)
C11 0.046(4) 0.042(4) 0.041(4) -0.002(3) -0.009(3) 0.000(3)
C12 0.049(4) 0.049(5) 0.056(5) -0.002(4) -0.003(4) -0.006(4)
C13 0.065(6) 0.099(8) 0.062(6) 0.004(5) -0.004(5) 0.024(5)
C14 0.078(6) 0.076(7) 0.073(6) 0.012(5) 0.024(5) -0.001(5)
C15 0.057(5) 0.047(5) 0.049(5) -0.006(4) 0.000(4) 0.004(4)
C16 0.072(6) 0.063(6) 0.058(5) 0.000(5) -0.011(4) 0.023(5)
C17 0.052(5) 0.063(6) 0.063(6) 0.003(4) 0.005(4) 0.005(4)
C18 0.040(4) 0.034(4) 0.040(4) 0.001(3) -0.006(3) 0.004(3)
C19 0.044(4) 0.039(4) 0.041(4) 0.005(3) -0.002(3) 0.003(3)
C20 0.047(4) 0.048(5) 0.040(4) -0.005(4) 0.003(3) 0.008(3)
C21 0.048(4) 0.048(5) 0.047(5) -0.009(4) -0.008(4) 0.002(4)

C22 0.041(4) 0.046(5) 0.050(5) 0.007(4) -0.007(4) -0.006(3)
C23 0.050(4) 0.040(5) 0.043(4) 0.004(3) -0.008(4) -0.003(3)
C24 0.043(4) 0.079(6) 0.053(5) -0.008(4) -0.001(4) -0.022(4)
C25 0.064(6) 0.115(9) 0.068(6) 0.022(6) 0.007(5) -0.021(6)
C26 0.054(5) 0.088(7) 0.082(7) -0.007(6) 0.007(5) -0.002(5)
C27 0.048(4) 0.065(6) 0.043(4) -0.006(4) 0.000(3) 0.001(4)
C28 0.059(5) 0.062(6) 0.059(5) -0.005(4) 0.003(4) -0.003(4)
C29 0.044(5) 0.078(6) 0.064(6) -0.001(5) 0.002(4) 0.002(4)
C30 0.110(8) 0.057(6) 0.057(5) 0.004(5) 0.023(6) 0.003(6)
C31 0.071(6) 0.058(6) 0.046(5) -0.001(4) -0.006(4) 0.000(4)
C32 0.050(5) 0.056(5) 0.058(5) -0.010(4) 0.001(4) -0.002(4)
C33 0.056(5) 0.071(6) 0.046(5) -0.002(4) 0.000(4) 0.003(4)
C34 0.048(5) 0.056(6) 0.080(7) 0.001(5) 0.000(4) 0.000(4)
C35 0.065(6) 0.057(6) 0.095(8) -0.002(5) 0.028(6) -0.005(4)
C36 0.072(7) 0.099(8) 0.071(6) -0.004(6) -0.001(5) -0.015(6)

_geom_special_details
;
 All s.u.'s (except the s.u. in the dihedral angle between two l.s. planes)
 are estimated using the full covariance matrix. The cell s.u.'s are taken
 into account individually in the estimation of s.u.'s in distances, angles
 and torsion angles; correlations between s.u.'s in cell parameters are only
 used when they are defined by crystal symmetry. An approximate (isotropic)
 treatment of cell s.u.'s is used for estimating s.u.'s involving l.s. planes.
;

loop_
 _geom_bond_atom_site_label_1
 _geom_bond_atom_site_label_2
 _geom_bond_distance
 _geom_bond_site_symmetry_2

_geom_bond_publ_flag
Zr1 N1 2.152(6) . ?
Zr1 N2 2.168(6) . ?
Zr1 Cl2 2.388(2) . ?
Zr1 Cl3 2.3935(19) . ?
Zr1 Cl1 2.584(2) . ?
Zr1 Cl1 2.596(2) 3 ?
Zr1 C1 2.674(7) . ?
Cl1 Zr1 2.596(2) 3 ?
N1 C1 1.376(9) . ?
N1 C6 1.439(9) . ?
N2 C1 1.347(9) . ?
N2 C18 1.458(9) . ?
C1 N3 1.331(9) . ?
N3 C4 1.479(10) . ?
N3 C2 1.479(8) . ?
C2 C3 1.519(12) . ?
C4 C5 1.495(12) . ?
C6 C7 1.403(11) . ?
C6 C11 1.406(11) . ?
C7 C8 1.403(11) . ?
C7 C15 1.537(11) . ?
C8 C9 1.372(12) . ?
C9 C10 1.376(12) . ?
C10 C11 1.376(10) . ?
C11 C12 1.511(11) . ?
C12 C13 1.522(11) . ?
C12 C14 1.537(12) . ?
C15 C16 1.519(11) . ?
C15 C17 1.526(12) . ?
C18 C19 1.398(10) . ?

C18 C23 1.441(10) . ?
C19 C20 1.384(10) . ?
C19 C27 1.525(11) . ?
C20 C21 1.373(11) . ?
C21 C22 1.368(11) . ?
C22 C23 1.379(11) . ?
C23 C24 1.515(12) . ?
C24 C25 1.510(13) . ?
C24 C26 1.565(12) . ?
C27 C28 1.526(12) . ?
C27 C29 1.538(11) . ?
C30 C31 1.355(14) . ?
C30 C35 1.384(14) . ?
C31 C32 1.392(12) . ?
C32 C33 1.415(11) . ?
C32 C36 1.484(13) . ?
C33 C34 1.348(12) . ?
C34 C35 1.378(14) . ?

loop_
 _geom_angle_atom_site_label_1
 _geom_angle_atom_site_label_2
 _geom_angle_atom_site_label_3
 _geom_angle
 _geom_angle_site_symmetry_1
 _geom_angle_site_symmetry_3
 _geom_angle_publ_flag
N1 Zr1 N2 60.7(2) . . ?
N1 Zr1 Cl2 105.12(18) . . ?
N2 Zr1 Cl2 96.40(16) . . ?
N1 Zr1 Cl3 87.42(16) . . ?

N2 Zr1 Cl3 147.88(16) . . ?

Cl2 Zr1 Cl3 95.29(8) . . ?

N1 Zr1 Cl1 95.59(18) . . ?

N2 Zr1 Cl1 91.85(16) . . ?

Cl2 Zr1 Cl1 159.19(7) . . ?

Cl3 Zr1 Cl1 87.60(7) . . ?

N1 Zr1 Cl1 157.43(17) . 3 ?

N2 Zr1 Cl1 98.44(16) . 3 ?

Cl2 Zr1 Cl1 84.43(7) . 3 ?

Cl3 Zr1 Cl1 112.41(7) . 3 ?

Cl1 Zr1 Cl1 75.46(7) . 3 ?

N1 Zr1 C1 30.8(2) . . ?

N2 Zr1 C1 30.0(2) . . ?

Cl2 Zr1 C1 100.14(16) . . ?

Cl3 Zr1 C1 118.20(16) . . ?

Cl1 Zr1 C1 96.56(16) . . ?

Cl1 Zr1 C1 128.36(16) 3 . ?

Zr1 Cl1 Zr1 104.54(7) . 3 ?

C1 N1 C6 128.6(6) . . ?

C1 N1 Zr1 96.1(4) . . ?

C6 N1 Zr1 133.8(5) . . ?

C1 N2 C18 126.7(6) . . ?

C1 N2 Zr1 96.3(4) . . ?

C18 N2 Zr1 132.2(4) . . ?

N3 C1 N2 126.6(6) . . ?

N3 C1 N1 126.7(6) . . ?

N2 C1 N1 106.5(6) . . ?

N3 C1 Zr1 170.8(5) . . ?

N2 C1 Zr1 53.7(3) . . ?

N1 C1 Zr1 53.2(4) . . ?

C1 N3 C4 122.8(6) . . ?

C1 N3 C2 119.3(6) . . ?

C4 N3 C2 116.2(6) . . ?

N3 C2 C3 111.2(6) . . ?

N3 C4 C5 114.0(7) . . ?

C7 C6 C11 120.8(7) . . ?

C7 C6 N1 120.8(7) . . ?

C11 C6 N1 118.3(7) . . ?

C8 C7 C6 118.3(7) . . ?

C8 C7 C15 118.1(7) . . ?

C6 C7 C15 123.6(7) . . ?

C9 C8 C7 120.8(8) . . ?

C8 C9 C10 120.0(8) . . ?

C11 C10 C9 121.9(8) . . ?

C10 C11 C6 118.3(7) . . ?

C10 C11 C12 119.2(7) . . ?

C6 C11 C12 122.5(7) . . ?

C11 C12 C13 112.6(7) . . ?

C11 C12 C14 112.8(7) . . ?

C13 C12 C14 110.2(7) . . ?

C16 C15 C17 111.6(7) . . ?

C16 C15 C7 112.7(7) . . ?

C17 C15 C7 109.5(6) . . ?

C19 C18 C23 120.1(7) . . ?

C19 C18 N2 121.6(6) . . ?

C23 C18 N2 118.1(6) . . ?

C20 C19 C18 118.2(7) . . ?

C20 C19 C27 118.3(7) . . ?

C18 C19 C27 123.5(7) . . ?

C21 C20 C19 122.4(7) . . ?

C22 C21 C20 119.4(7) . . ?

C21 C22 C23 122.1(7) . . ?

C22 C23 C18 117.8(7) . . ?

C22 C23 C24 119.4(7) . . ?

C18 C23 C24 122.8(7) . . ?

C25 C24 C23 112.8(8) . . ?

C25 C24 C26 109.5(7) . . ?

C23 C24 C26 108.8(7) . . ?

C19 C27 C28 109.1(6) . . ?

C19 C27 C29 113.1(7) . . ?

C28 C27 C29 109.8(7) . . ?

C31 C30 C35 121.6(9) . . ?

C30 C31 C32 121.5(8) . . ?

C31 C32 C33 116.5(8) . . ?

C31 C32 C36 122.5(8) . . ?

C33 C32 C36 121.0(8) . . ?

C34 C33 C32 120.8(9) . . ?

C33 C34 C35 122.3(8) . . ?

C34 C35 C30 117.3(9) . . ?

_diffrn_measured_fraction_theta_max   0.992

_diffrn_reflns_theta_full   25.69

_diffrn_measured_fraction_theta_full   0.992

_refine_diff_density_max   2.020

_refine_diff_density_min   -0.628

_refine_diff_density_rms   0.189

checkCIF/PLATON report

Structure factors have been supplied for datablock(s) j75mp-neu

THIS REPORT IS FOR GUIDANCE ONLY. IF USED AS PART OF A REVIEW PROCEDURE FOR PUBLICATION, IT SHOULD NOT REPLACE THE EXPERTISE OF AN EXPERIENCED CRYSTALLOGRAPHIC REFEREE.

No syntax errors found. CIF dictionary Interpreting this report

Datablock: j75mp-neu

```
Bond precision:   C-C = 0.0120 A          Wavelength=0.71069

Cell:            a=10.9383(10)     b=20.6555(18)       c=16.4615(16)
                 alpha=90          beta=100.105(5)     gamma=90
Temperature:     133 K

                 Calculated                      Reported
Volume           3661.6(6)                       3661.6(6)
Space group      P 21/n                          P21/n
Hall group       -P 2yn                          ?
                 C58 H88 Cl6 N6 Zr2, 2(C7
Moiety formula   H8)                             C36 H52 Cl3 N3 Zr
Sum formula      C72 H104 Cl6 N6 Zr2             C36 H52 Cl3 N3 Zr
Mr               1448.75                         724.38
Dx,g cm-3        1.314                           1.314
Z                2                               4
Mu (mm-1)        0.547                           0.547
F000             1520.0                          1520.0
F000'            1510.16
h,k,lmax         13,25,20                        13,25,20
Nref             6966                            6909
Tmin,Tmax        0.883,0.991                     0.853,0.949
Tmin'            0.864

Correction method= NUMERICAL

Data completeness= 0.992          Theta(max)= 25.690

R(reflections)= 0.0872( 3487)     wR2(reflections)= 0.2158( 6909)

S = 0.914                  Npar= 399
```

The following ALERTS were generated. Each ALERT has the format
test-name_ALERT_alert-type_alert-level.
Click on the hyperlinks for more details of the test.

```
Alert level B
RINTA01_ALERT_3_B  The value of Rint is greater than 0.18
            Rint given    0.247
PLAT020_ALERT_3_B The value of Rint is greater than 0.12 .........      0.247
PLAT413_ALERT_2_B Short Inter XH3 .. XHn      H25B    ..  H25B    ..     2.02 Ang.

Alert level C
ABSTY02_ALERT_1_C  An _exptl_absorpt_correction_type has been given without
            a literature citation. This should be contained in the
            _exptl_absorpt_process_details field.
            Absorption correction given as numerical
PLAT094_ALERT_2_C Ratio of Maximum / Minimum Residual Density ....       3.22
PLAT342_ALERT_3_C Low Bond Precision on  C-C Bonds ..............     0.0120 Ang.

Alert level G
PLAT005_ALERT_5_G No _iucr_refine_instructions_details  in the CIF          ? Do !
PLAT042_ALERT_1_G Calc. and Reported MoietyFormula Strings  Differ          ? Check
PLAT045_ALERT_1_G Calculated and Reported Z Differ by ............       0.50 Ratio
PLAT072_ALERT_2_G SHELXL First  Parameter in WGHT Unusually Large.       0.11
PLAT128_ALERT_4_G Note: Alternate Setting of Space-group P21/c    .      P21/n
PLAT790_ALERT_4_G Centre of Gravity not Within Unit Cell: Resd.  #          2
              C7 H8
PLAT794_ALERT_5_G Note: Tentative Bond Valency for Zr1     (IV)          4.61

   0 ALERT level A = Most likely a serious problem - resolve or explain
   3 ALERT level B = A potentially serious problem, consider carefully
   3 ALERT level C = Check. Ensure it is not caused by an omission or oversight
   7 ALERT level G = General information/check it is not something unexpected

   3 ALERT type 1 CIF construction/syntax error, inconsistent or missing data
   3 ALERT type 2 Indicator that the structure model may be wrong or deficient
   3 ALERT type 3 Indicator that the structure quality may be low
   2 ALERT type 4 Improvement, methodology, query or suggestion
   2 ALERT type 5 Informative message, check
```

It is advisable to attempt to resolve as many as possible of the alerts in all categories. Often the minor alerts point to easily fixed oversights, errors and omissions in your CIF or refinement strategy, so attention to these fine details can be worthwhile. In order to resolve some of the more serious problems it may be necessary to carry out additional measurements or structure refinements. However, the purpose of your study may justify the reported deviations and the more serious of these should normally be commented upon in the discussion or experimental section of a paper or in the "special_details" fields of the CIF. checkCIF was carefully designed to identify outliers and unusual parameters, but every test has its limitations and alerts that are not important in a particular case may appear. Conversely, the absence of alerts does not guarantee there are no aspects of the results needing attention. It is up to the individual to critically assess their own results and, if necessary, seek expert advice.

Publication of your CIF in IUCr journals

A basic structural check has been run on your CIF. These basic checks will be run on all CIFs submitted for publication in IUCr journals (*Acta Crystallographica*, *Journal of Applied Crystallography*, *Journal of Synchrotron Radiation*); however, if you intend to submit to *Acta Crystallographica Section C* or *E*, you should make sure that full publication checks are run on the final version of your CIF prior to submission.

Publication of your CIF in other journals

Please refer to the *Notes for Authors* of the relevant journal for any special instructions relating to CIF submission.

PLATON version of 01/06/2013; check.def file version of 24/05/2013

Datablock j75mp-neu - ellipsoid plot

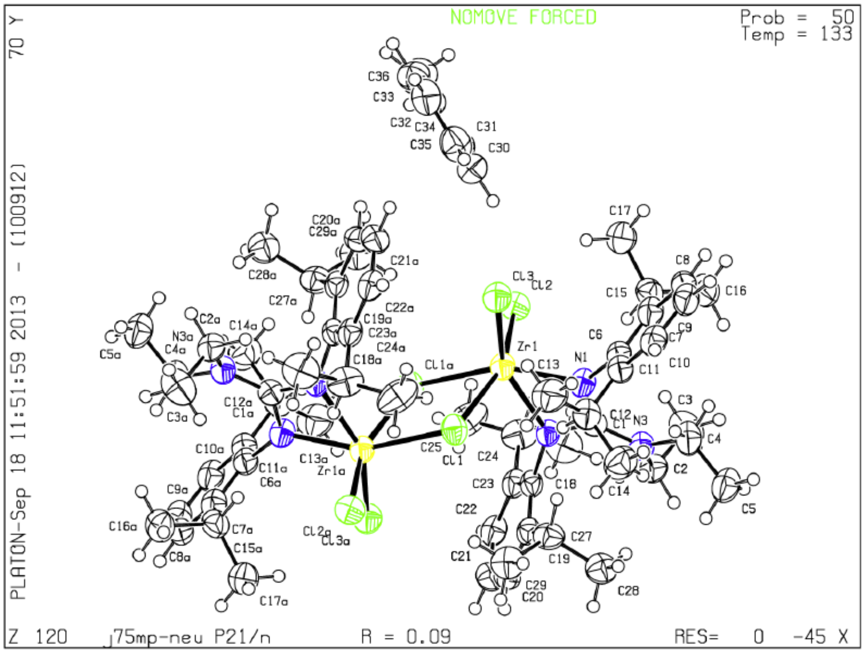

Fig. S 5. Check CIF compound 0

CIF File coumpound **I**:

```
data_j115mp-p21

_audit_creation_method            SHELXL-97
_chemical_name_systematic
;
 ?
;
_chemical_name_common             ?
_chemical_melting_point           ?
_chemical_formula_moiety          ?
_chemical_formula_sum
 'C32 H53 N3 Zr'
_chemical_formula_weight          570.99

loop_
 _atom_type_symbol
 _atom_type_description
 _atom_type_scat_dispersion_real
 _atom_type_scat_dispersion_imag
 _atom_type_scat_source
 'C'  'C'   0.0033   0.0016
 'International Tables Vol C Tables 4.2.6.8 and 6.1.1.4'
 'H'  'H'   0.0000   0.0000
 'International Tables Vol C Tables 4.2.6.8 and 6.1.1.4'
 'N'  'N'   0.0061   0.0033
 'International Tables Vol C Tables 4.2.6.8 and 6.1.1.4'
 'Zr'  'Zr'  -2.9673   0.5597
 'International Tables Vol C Tables 4.2.6.8 and 6.1.1.4'
```

```
_symmetry_cell_setting            monoclinic
_symmetry_space_group_name_H-M    P2(1)

loop_
 _symmetry_equiv_pos_as_xyz
 'x, y, z'
 '-x, y+1/2, -z'

_cell_length_a                    9.4553(4)
_cell_length_b                    17.2321(7)
_cell_length_c                    10.3607(4)
_cell_angle_alpha                 90.00
_cell_angle_beta                  108.682(3)
_cell_angle_gamma                 90.00
_cell_volume                      1599.17(11)
_cell_formula_units_Z             2
_cell_measurement_temperature     133(2)
_cell_measurement_reflns_used     all
_cell_measurement_theta_min       2.08
_cell_measurement_theta_max       27.11

_exptl_crystal_description        block
_exptl_crystal_colour             colourless
_exptl_crystal_size_max           0.177
_exptl_crystal_size_mid           0.159
_exptl_crystal_size_min           0.147
_exptl_crystal_density_meas       ?
_exptl_crystal_density_diffrn     1.186
_exptl_crystal_density_method     'not measured'
_exptl_crystal_F_000              612
_exptl_absorpt_coefficient_mu     0.366
```

```
_exptl_absorpt_correction_type    numerical
_exptl_absorpt_correction_T_min   0.9241
_exptl_absorpt_correction_T_max   0.9696
_exptl_absorpt_process_details    ?

_exptl_special_details
;
 ?
;

_diffrn_ambient_temperature       133(2)
_diffrn_radiation_wavelength      0.71069
_diffrn_radiation_type            MoK\a
_diffrn_radiation_source          'fine-focus sealed tube'
_diffrn_radiation_monochromator   graphite
_diffrn_measurement_device_type   STOE-IPDS-II
_diffrn_measurement_method        omega-scan
_diffrn_detector_area_resol_mean  ?
_diffrn_standards_number          ?
_diffrn_standards_interval_count  ?
_diffrn_standards_interval_time   ?
_diffrn_standards_decay_%         ?
_diffrn_reflns_number             23605
_diffrn_reflns_av_R_equivalents   0.0645
_diffrn_reflns_av_sigmaI/netI     0.0742
_diffrn_reflns_limit_h_min        -11
_diffrn_reflns_limit_h_max        11
_diffrn_reflns_limit_k_min        -21
_diffrn_reflns_limit_k_max        21
_diffrn_reflns_limit_l_min        -13
_diffrn_reflns_limit_l_max        13
```

_diffrn_reflns_theta_min 2.07
_diffrn_reflns_theta_max 26.76
_reflns_number_total 6693
_reflns_number_gt 5468
_reflns_threshold_expression >2\s(I)

_computing_data_collection STOE-XAREA
_computing_cell_refinement STOE-XAREA
_computing_data_reduction STOE-XAREA
_computing_structure_solution Sor-97
_computing_structure_refinement 'SHELXL-97 (Sheldrick, 2008)'
_computing_molecular_graphics ?
_computing_publication_material ?

_refine_special_details
;
Refinement of F^2^ against ALL reflections. The weighted R-factor wR and goodness of fit S are based on F^2^, conventional R-factors R are based on F, with F set to zero for negative F^2^. The threshold expression of F^2^ > 2\s(F^2^) is used only for calculating R-factors(gt) etc. and is not relevant to the choice of reflections for refinement. R-factors based on F^2^ are statistically about twice as large as those based on F, and R-factors based on ALL data will be even larger.
;

_refine_ls_structure_factor_coef Fsqd
_refine_ls_matrix_type full
_refine_ls_weighting_scheme calc
_refine_ls_weighting_details
'calc w=1/[\s^2^(Fo^2^)+(0.0314P)^2^+0.0000P] where P=(Fo^2^+2Fc^2^)/3'
_atom_sites_solution_primary direct

```
_atom_sites_solution_secondary    difmap
_atom_sites_solution_hydrogens    geom
_refine_ls_hydrogen_treatment     constr
_refine_ls_extinction_method      none
_refine_ls_extinction_coef        ?
_refine_ls_abs_structure_details
 'Flack H D (1983), Acta Cryst. A39, 876-881'
_refine_ls_abs_structure_Flack    0.02(3)
_refine_ls_number_reflns          6693
_refine_ls_number_parameters      338
_refine_ls_number_restraints      1
_refine_ls_R_factor_all           0.0531
_refine_ls_R_factor_gt            0.0379
_refine_ls_wR_factor_ref          0.0724
_refine_ls_wR_factor_gt           0.0695
_refine_ls_goodness_of_fit_ref    0.921
_refine_ls_restrained_S_all       0.921
_refine_ls_shift/su_max           0.018
_refine_ls_shift/su_mean          0.001

loop_
 _atom_site_label
 _atom_site_type_symbol
 _atom_site_fract_x
 _atom_site_fract_y
 _atom_site_fract_z
 _atom_site_U_iso_or_equiv
 _atom_site_adp_type
 _atom_site_occupancy
 _atom_site_symmetry_multiplicity
 _atom_site_calc_flag
```

_atom_site_refinement_flags
_atom_site_disorder_assembly
_atom_site_disorder_group
C1 C 1.1796(3) 0.53239(18) 0.6697(3) 0.0195(7) Uani 1 1 d . . .
C2 C 1.0338(3) 0.65333(17) 0.6605(3) 0.0228(8) Uani 1 1 d . . .
C3 C 0.9521(3) 0.65768(18) 0.5197(3) 0.0264(7) Uani 1 1 d . . .
C4 C 0.8232(4) 0.7027(2) 0.4818(4) 0.0342(8) Uani 1 1 d . . .
H4 H 0.7647 0.7051 0.3883 0.041 Uiso 1 1 calc R . .
C5 C 0.7777(4) 0.7436(2) 0.5744(4) 0.0390(9) Uani 1 1 d . . .
H5 H 0.6873 0.7724 0.5461 0.047 Uiso 1 1 calc R . .
C6 C 0.8650(4) 0.7426(2) 0.7093(4) 0.0342(9) Uani 1 1 d . . .
H6 H 0.8360 0.7733 0.7728 0.041 Uiso 1 1 calc R . .
C7 C 0.9931(4) 0.6984(2) 0.7554(3) 0.0272(8) Uani 1 1 d . . .
C8 C 1.0884(4) 0.6989(2) 0.9051(3) 0.0330(8) Uani 1 1 d . . .
H8 H 1.1946 0.6968 0.9065 0.040 Uiso 1 1 calc R . .
C9 C 1.0646(4) 0.6283(5) 0.9794(3) 0.0527(9) Uani 1 1 d . . .
H9A H 1.1361 0.6283 1.0719 0.079 Uiso 1 1 calc R . .
H9B H 1.0795 0.5817 0.9310 0.079 Uiso 1 1 calc R . .
H9C H 0.9627 0.6286 0.9840 0.079 Uiso 1 1 calc R . .
C10 C 1.0741(5) 0.7717(3) 0.9806(5) 0.0668(15) Uani 1 1 d . . .
H10A H 0.9743 0.7739 0.9901 0.100 Uiso 1 1 calc R . .
H10B H 1.0890 0.8172 0.9296 0.100 Uiso 1 1 calc R . .
H10C H 1.1495 0.7714 1.0711 0.100 Uiso 1 1 calc R . .
C11 C 1.0086(3) 0.6231(4) 0.4109(3) 0.0298(7) Uani 1 1 d . . .
H11 H 1.0656 0.5750 0.4498 0.036 Uiso 1 1 calc R . .
C12 C 0.8849(4) 0.5997(2) 0.2817(4) 0.0473(11) Uani 1 1 d . . .
H12A H 0.8098 0.5690 0.3058 0.071 Uiso 1 1 calc R . .
H12B H 0.9273 0.5687 0.2238 0.071 Uiso 1 1 calc R . .
H12C H 0.8381 0.6464 0.2323 0.071 Uiso 1 1 calc R . .
C13 C 1.1171(4) 0.6789(2) 0.3792(4) 0.0426(10) Uani 1 1 d . . .
H13A H 1.1606 0.6545 0.3152 0.064 Uiso 1 1 calc R . .

H13B H 1.1967 0.6921 0.4636 0.064 Uiso 1 1 calc R . .

H13C H 1.0640 0.7263 0.3384 0.064 Uiso 1 1 calc R . .

C14 C 1.3975(3) 0.45233(19) 0.6647(3) 0.0231(7) Uani 1 1 d . . .

C15 C 1.4462(3) 0.44372(19) 0.5498(3) 0.0258(7) Uani 1 1 d . . .

C16 C 1.5342(3) 0.3812(2) 0.5444(4) 0.0311(8) Uani 1 1 d . . .

H16 H 1.5682 0.3755 0.4680 0.037 Uiso 1 1 calc R . .

C17 C 1.5743(4) 0.3269(2) 0.6464(4) 0.0355(8) Uani 1 1 d . . .

H17 H 1.6353 0.2841 0.6404 0.043 Uiso 1 1 calc R . .

C18 C 1.5263(4) 0.3346(2) 0.7563(4) 0.0336(8) Uani 1 1 d . . .

H18 H 1.5529 0.2962 0.8257 0.040 Uiso 1 1 calc R . .

C19 C 1.4395(3) 0.39724(19) 0.7699(3) 0.0270(7) Uani 1 1 d . . .

C20 C 1.4002(4) 0.4063(2) 0.9007(4) 0.0349(8) Uani 1 1 d . . .

H20 H 1.3343 0.4528 0.8906 0.042 Uiso 1 1 calc R . .

C21 C 1.5411(5) 0.4204(2) 1.0212(4) 0.0440(10) Uani 1 1 d . . .

H21A H 1.6015 0.3730 1.0405 0.066 Uiso 1 1 calc R . .

H21B H 1.5134 0.4346 1.1015 0.066 Uiso 1 1 calc R . .

H21C H 1.5990 0.4626 0.9991 0.066 Uiso 1 1 calc R . .

C22 C 1.3163(4) 0.3362(2) 0.9291(4) 0.0439(10) Uani 1 1 d . . .

H22A H 1.2264 0.3272 0.8510 0.066 Uiso 1 1 calc R . .

H22B H 1.2878 0.3460 1.0106 0.066 Uiso 1 1 calc R . .

H22C H 1.3808 0.2903 0.9438 0.066 Uiso 1 1 calc R . .

C23 C 1.4007(4) 0.5010(2) 0.4320(3) 0.0317(8) Uani 1 1 d . . .

H23 H 1.3490 0.5455 0.4600 0.038 Uiso 1 1 calc R . .

C24 C 1.5330(4) 0.5335(3) 0.3967(4) 0.0466(10) Uani 1 1 d . . .

H24A H 1.6007 0.5598 0.4765 0.070 Uiso 1 1 calc R . .

H24B H 1.4976 0.5705 0.3215 0.070 Uiso 1 1 calc R . .

H24C H 1.5861 0.4910 0.3694 0.070 Uiso 1 1 calc R . .

C25 C 1.2898(4) 0.4637(3) 0.3062(4) 0.0476(10) Uani 1 1 d . . .

H25A H 1.3389 0.4212 0.2743 0.071 Uiso 1 1 calc R . .

H25B H 1.2552 0.5027 0.2340 0.071 Uiso 1 1 calc R . .

H25C H 1.2044 0.4434 0.3296 0.071 Uiso 1 1 calc R . .

C26 C 1.0757(4) 0.4083(2) 0.5647(4) 0.0275(8) Uani 1 1 d . . .

H26A H 1.0991 0.3650 0.6309 0.033 Uiso 1 1 calc R . .

H26B H 1.1590 0.4126 0.5266 0.033 Uiso 1 1 calc R . .

C27 C 0.9339(4) 0.3890(3) 0.4502(5) 0.0441(11) Uani 1 1 d . . .

H27A H 0.8539 0.3773 0.4883 0.066 Uiso 1 1 calc R . .

H27B H 0.9513 0.3438 0.3999 0.066 Uiso 1 1 calc R . .

H27C H 0.9048 0.4334 0.3883 0.066 Uiso 1 1 calc R . .

C28 C 0.9374(4) 0.48820(19) 0.6845(3) 0.0267(7) Uani 1 1 d . . .

H28A H 0.8451 0.4833 0.6055 0.032 Uiso 1 1 calc R . .

H28B H 0.9379 0.5406 0.7240 0.032 Uiso 1 1 calc R . .

C29 C 0.9350(4) 0.4280(2) 0.7900(4) 0.0410(9) Uani 1 1 d . . .

H29A H 0.9353 0.3760 0.7519 0.061 Uiso 1 1 calc R . .

H29B H 0.8447 0.4347 0.8159 0.061 Uiso 1 1 calc R . .

H29C H 1.0234 0.4343 0.8706 0.061 Uiso 1 1 calc R . .

C30 C 1.4597(3) 0.6265(4) 1.0218(3) 0.0366(7) Uani 1 1 d . . .

H30A H 1.4460 0.6785 1.0545 0.055 Uiso 1 1 calc R . .

H30B H 1.5634 0.6101 1.0642 0.055 Uiso 1 1 calc R . .

H30C H 1.3929 0.5899 1.0460 0.055 Uiso 1 1 calc R . .

C31 C 1.4076(5) 0.7504(2) 0.7208(4) 0.0349(9) Uani 1 1 d . . .

H31A H 1.4203 0.7497 0.6306 0.052 Uiso 1 1 calc R . .

H31B H 1.4902 0.7791 0.7844 0.052 Uiso 1 1 calc R . .

H31C H 1.3129 0.7758 0.7147 0.052 Uiso 1 1 calc R . .

C32 C 1.6305(3) 0.5915(2) 0.7831(4) 0.0291(8) Uani 1 1 d . . .

H32A H 1.6516 0.5384 0.8178 0.044 Uiso 1 1 calc R . .

H32B H 1.7082 0.6264 0.8383 0.044 Uiso 1 1 calc R . .

H32C H 1.6290 0.5933 0.6882 0.044 Uiso 1 1 calc R . .

N1 N 1.3213(3) 0.52147(16) 0.6743(3) 0.0212(6) Uani 1 1 d . . .

N2 N 1.1649(3) 0.60703(13) 0.7079(2) 0.0214(7) Uani 1 1 d . . .

N3 N 1.0671(3) 0.48059(15) 0.6371(3) 0.0238(6) Uani 1 1 d . . .

Zr1 Zr 1.40608(3) 0.629168(18) 0.79498(3) 0.02342(7) Uani 1 1 d . . .

```
loop_
 _atom_site_aniso_label
 _atom_site_aniso_U_11
 _atom_site_aniso_U_22
 _atom_site_aniso_U_33
 _atom_site_aniso_U_23
 _atom_site_aniso_U_13
 _atom_site_aniso_U_12
C1 0.0272(16) 0.0187(18) 0.0135(14) 0.0015(13) 0.0077(12) 0.0004(13)
C2 0.0241(16) 0.019(2) 0.0285(16) 0.0041(12) 0.0126(13) -0.0015(11)
C3 0.0234(16) 0.0251(18) 0.0292(17) 0.0052(13) 0.0061(13) -0.0043(12)
C4 0.0268(18) 0.033(2) 0.039(2) 0.0121(17) 0.0055(16) -0.0008(15)
C5 0.0280(19) 0.034(2) 0.056(3) 0.0155(19) 0.0142(18) 0.0097(16)
C6 0.036(2) 0.024(2) 0.048(2) 0.0012(17) 0.0200(19) 0.0041(16)
C7 0.0280(17) 0.0234(19) 0.0331(19) 0.0000(15) 0.0136(15) 0.0000(14)
C8 0.0298(18) 0.037(2) 0.0332(19) -0.0093(17) 0.0118(16) 0.0011(16)
C9 0.053(2) 0.076(3) 0.0263(15) -0.001(4) 0.0101(14) -0.008(4)
C10 0.055(3) 0.076(4) 0.062(3) -0.035(3) 0.008(2) 0.013(3)
C11 0.0312(14) 0.033(2) 0.0225(13) 0.008(2) 0.0053(11) -0.002(2)
C12 0.041(2) 0.062(3) 0.036(2) -0.0077(19) 0.0085(17) -0.0094(18)
C13 0.047(2) 0.048(3) 0.036(2) -0.0032(19) 0.0182(18) -0.0126(19)
C14 0.0187(15) 0.0217(18) 0.0292(17) -0.0013(14) 0.0079(13) -0.0016(13)
C15 0.0245(16) 0.0233(18) 0.0316(18) -0.0078(14) 0.0116(14) -0.0068(13)
C16 0.027(2) 0.031(2) 0.038(2) -0.0082(19) 0.0134(15) 0.0003(16)
C17 0.0300(18) 0.029(2) 0.048(2) -0.0094(18) 0.0137(16) 0.0026(15)
C18 0.0330(19) 0.025(2) 0.041(2) 0.0027(17) 0.0099(16) 0.0026(15)
C19 0.0262(17) 0.0238(19) 0.0315(19) 0.0025(15) 0.0101(14) 0.0009(13)
C20 0.045(2) 0.026(2) 0.036(2) 0.0070(17) 0.0159(17) 0.0093(17)
C21 0.058(3) 0.041(2) 0.032(2) 0.0022(18) 0.0129(18) 0.0007(19)
C22 0.043(2) 0.052(3) 0.042(2) 0.016(2) 0.0206(19) 0.0094(19)
C23 0.0324(18) 0.037(2) 0.0299(18) 0.0021(16) 0.0159(15) 0.0048(15)
```

C24 0.050(2) 0.053(3) 0.038(2) 0.0082(19) 0.0147(18) -0.009(2)
C25 0.041(2) 0.064(3) 0.035(2) 0.007(2) 0.0078(18) -0.008(2)
C26 0.0278(18) 0.022(2) 0.034(2) -0.0039(17) 0.0119(15) -0.0016(16)
C27 0.033(2) 0.045(3) 0.054(3) -0.019(2) 0.0136(18) -0.004(2)
C28 0.0265(17) 0.0227(19) 0.0334(19) 0.0006(15) 0.0131(15) -0.0007(14)
C29 0.044(2) 0.035(2) 0.054(2) 0.008(2) 0.030(2) 0.0032(17)
C30 0.0391(15) 0.0367(18) 0.0302(14) -0.001(3) 0.0059(12) 0.004(3)
C31 0.045(2) 0.026(2) 0.0267(19) 0.0047(16) 0.0025(17) -0.0045(17)
C32 0.0234(16) 0.0265(18) 0.0352(19) -0.0044(15) 0.0064(14) -0.0032(13)
N1 0.0222(14) 0.0233(16) 0.0196(14) 0.0016(12) 0.0088(11) 0.0001(12)
N2 0.0231(13) 0.021(2) 0.0207(12) 0.0012(10) 0.0077(10) 0.0011(10)
N3 0.0254(14) 0.0211(15) 0.0274(14) -0.0030(12) 0.0118(11) -0.0007(11)
Zr1 0.02317(12) 0.02077(13) 0.02546(13) -0.0007(3) 0.00656(9) -0.0017(2)

_geom_special_details
;
All s.u.'s (except the s.u. in the dihedral angle between two l.s. planes)
are estimated using the full covariance matrix. The cell s.u.'s are taken
into account individually in the estimation of s.u.'s in distances, angles
and torsion angles; correlations between s.u.'s in cell parameters are only
used when they are defined by crystal symmetry. An approximate (isotropic)
treatment of cell s.u.'s is used for estimating s.u.'s involving l.s. planes.
;

loop_
_geom_bond_atom_site_label_1
_geom_bond_atom_site_label_2
_geom_bond_distance
_geom_bond_site_symmetry_2
_geom_bond_publ_flag
C1 N1 1.338(4) . ?

C1 N3 1.346(4) . ?

C1 N2 1.366(4) . ?

C1 Zr1 2.697(3) . ?

C2 C7 1.401(5) . ?

C2 C3 1.418(4) . ?

C2 N2 1.422(4) . ?

C3 C4 1.391(5) . ?

C3 C11 1.516(5) . ?

C4 C5 1.366(5) . ?

C5 C6 1.377(5) . ?

C6 C7 1.379(5) . ?

C7 C8 1.524(5) . ?

C8 C9 1.494(8) . ?

C8 C10 1.508(5) . ?

C11 C13 1.518(6) . ?

C11 C12 1.523(5) . ?

C14 C19 1.404(4) . ?

C14 N1 1.413(4) . ?

C14 C15 1.415(4) . ?

C15 C16 1.373(5) . ?

C15 C23 1.521(5) . ?

C16 C17 1.372(5) . ?

C17 C18 1.361(5) . ?

C18 C19 1.390(5) . ?

C19 C20 1.523(5) . ?

C20 C22 1.525(5) . ?

C20 C21 1.525(5) . ?

C23 C24 1.518(5) . ?

C23 C25 1.528(5) . ?

C26 N3 1.470(4) . ?

C26 C27 1.516(5) . ?

C28 N3 1.466(4) . ?

C28 C29 1.513(5) . ?

C30 Zr1 2.240(3) . ?

C31 Zr1 2.228(4) . ?

C32 Zr1 2.260(3) . ?

N1 Zr1 2.238(3) . ?

N2 Zr1 2.201(2) . ?

loop_
 _geom_angle_atom_site_label_1
 _geom_angle_atom_site_label_2
 _geom_angle_atom_site_label_3
 _geom_angle
 _geom_angle_site_symmetry_1
 _geom_angle_site_symmetry_3
 _geom_angle_publ_flag
N1 C1 N3 128.1(3) . . ?
N1 C1 N2 108.4(3) . . ?
N3 C1 N2 123.4(3) . . ?
N1 C1 Zr1 55.82(16) . . ?
N3 C1 Zr1 166.6(2) . . ?
N2 C1 Zr1 54.35(15) . . ?
C7 C2 C3 120.8(3) . . ?
C7 C2 N2 118.3(3) . . ?
C3 C2 N2 120.8(3) . . ?
C4 C3 C2 117.3(3) . . ?
C4 C3 C11 119.7(3) . . ?
C2 C3 C11 122.6(3) . . ?
C5 C4 C3 122.3(3) . . ?
C4 C5 C6 119.0(3) . . ?
C5 C6 C7 122.2(4) . . ?

C6 C7 C2 118.1(3) . . ?

C6 C7 C8 121.1(3) . . ?

C2 C7 C8 120.7(3) . . ?

C9 C8 C10 110.9(4) . . ?

C9 C8 C7 112.5(3) . . ?

C10 C8 C7 114.2(3) . . ?

C3 C11 C13 109.5(4) . . ?

C3 C11 C12 113.8(3) . . ?

C13 C11 C12 111.1(3) . . ?

C19 C14 N1 122.6(3) . . ?

C19 C14 C15 119.7(3) . . ?

N1 C14 C15 117.3(3) . . ?

C16 C15 C14 118.8(3) . . ?

C16 C15 C23 119.8(3) . . ?

C14 C15 C23 121.5(3) . . ?

C17 C16 C15 121.7(3) . . ?

C18 C17 C16 119.6(3) . . ?

C17 C18 C19 121.8(3) . . ?

C18 C19 C14 118.4(3) . . ?

C18 C19 C20 119.3(3) . . ?

C14 C19 C20 122.2(3) . . ?

C19 C20 C22 112.4(3) . . ?

C19 C20 C21 110.3(3) . . ?

C22 C20 C21 109.9(3) . . ?

C24 C23 C15 112.9(3) . . ?

C24 C23 C25 110.0(3) . . ?

C15 C23 C25 110.6(3) . . ?

N3 C26 C27 113.9(3) . . ?

N3 C28 C29 112.9(3) . . ?

C1 N1 C14 130.1(3) . . ?

C1 N1 Zr1 94.53(19) . . ?

C14 N1 Zr1 129.7(2) . . ?

C1 N2 C2 125.9(2) . . ?

C1 N2 Zr1 95.36(18) . . ?

C2 N2 Zr1 135.89(18) . . ?

C1 N3 C28 122.4(3) . . ?

C1 N3 C26 121.4(3) . . ?

C28 N3 C26 115.9(3) . . ?

N2 Zr1 C31 98.13(12) . . ?

N2 Zr1 N1 59.23(9) . . ?

C31 Zr1 N1 128.73(12) . . ?

N2 Zr1 C30 106.43(11) . . ?

C31 Zr1 C30 110.9(2) . . ?

N1 Zr1 C30 119.30(19) . . ?

N2 Zr1 C32 142.74(11) . . ?

C31 Zr1 C32 98.10(14) . . ?

N1 Zr1 C32 84.61(11) . . ?

C30 Zr1 C32 98.65(13) . . ?

N2 Zr1 C1 30.29(9) . . ?

C31 Zr1 C1 120.38(12) . . ?

N1 Zr1 C1 29.65(9) . . ?

C30 Zr1 C1 111.74(15) . . ?

C32 Zr1 C1 114.26(11) . . ?

_diffrn_measured_fraction_theta_max 0.997

_diffrn_reflns_theta_full 26.76

_diffrn_measured_fraction_theta_full 0.997

_refine_diff_density_max 0.571

_refine_diff_density_min -0.606

_refine_diff_density_rms 0.063

checkCIF/PLATON report

Structure factors have been supplied for datablock(s) j115mp-p21

THIS REPORT IS FOR GUIDANCE ONLY. IF USED AS PART OF A REVIEW PROCEDURE FOR PUBLICATION, IT SHOULD NOT REPLACE THE EXPERTISE OF AN EXPERIENCED CRYSTALLOGRAPHIC REFEREE.

No syntax errors found. CIF dictionary Interpreting this report

Datablock: j115mp-p21

```
Bond precision:   C-C = 0.0052 A          Wavelength=0.71069

Cell:             a=9.4553(4)     b=17.2321(7)        c=10.3607(4)
                  alpha=90        beta=108.682(3)     gamma=90
Temperature:      133 K

                 Calculated                 Reported
Volume           1599.17(12)                1599.17(11)
Space group      P 21                       P2(1)
Hall group       P 2yb                      ?
Moiety formula   C32 H53 N3 Zr              ?
Sum formula      C32 H53 N3 Zr              C32 H53 N3 Zr
Mr               570.99                     570.99
Dx,g cm-3        1.186                      1.186
Z                2                          2
Mu (mm-1)        0.366                      0.366
F000             612.0                      612.0
F000'            606.16
h,k,lmax         11,21,13                   11,21,13
Nref             6805[ 3519]                6693
Tmin,Tmax        0.937,0.948                0.924,0.970
Tmin'            0.937

Correction method= NUMERICAL

Data completeness= 1.90/0.98       Theta(max)= 26.760

R(reflections)= 0.0379( 5468)      wR2(reflections)= 0.0724( 6693)

S = 0.921                  Npar= Npar = 338
```

The following ALERTS were generated. Each ALERT has the format
test-name_ALERT_alert-type_alert-level.
Click on the hyperlinks for more details of the test.

Alert level C

```
ABSTY02_ALERT_1_C  An _exptl_absorpt_correction_type has been given without
            a literature citation. This should be contained in the
            _exptl_absorpt_process_details field.
            Absorption correction given as numerical
PLAT220_ALERT_2_C Large Non-Solvent  C     Ueq(max)/Ueq(min) Range       3.4 Ratio
PLAT242_ALERT_2_C Low       Ueq as Compared to Neighbors for .....        C8 Check
PLAT790_ALERT_4_C Centre of Gravity not Within Unit Cell: Resd.  #         1 Note
              C32 H53 N3 Zr
```

Alert level G

```
PLAT005_ALERT_5_G No _iucr_refine_instructions_details  in the CIF     Please Do !
PLAT794_ALERT_5_G Tentative Bond Valency for Zr1     (IV)     .....      3.35 Note
```

```
0 ALERT level A = Most likely a serious problem - resolve or explain
0 ALERT level B = A potentially serious problem, consider carefully
4 ALERT level C = Check. Ensure it is not caused by an omission or oversight
2 ALERT level G = General information/check it is not something unexpected

1 ALERT type 1 CIF construction/syntax error, inconsistent or missing data
2 ALERT type 2 Indicator that the structure model may be wrong or deficient
0 ALERT type 3 Indicator that the structure quality may be low
1 ALERT type 4 Improvement, methodology, query or suggestion
2 ALERT type 5 Informative message, check
```

It is advisable to attempt to resolve as many as possible of the alerts in all categories. Often the minor alerts point to easily fixed oversights, errors and omissions in your CIF or refinement strategy, so attention to these fine details can be worthwhile. In order to resolve some of the more serious problems it may be necessary to carry out additional measurements or structure refinements. However, the purpose of your study may justify the reported deviations and the more serious of these should normally be commented upon in the discussion or experimental section of a paper or in the "special_details" fields of the CIF. checkCIF was carefully designed to identify outliers and unusual parameters, but every test has its limitations and alerts that are not important in a particular case may appear. Conversely, the absence of alerts does not guarantee there are no aspects of the results needing attention. It is up to the individual to critically assess their own results and, if necessary, seek expert advice.

Publication of your CIF in IUCr journals

A basic structural check has been run on your CIF. These basic checks will be run on all CIFs submitted for publication in IUCr journals (*Acta Crystallographica*, *Journal of Applied Crystallography*, *Journal of Synchrotron Radiation*); however, if you intend to submit to *Acta Crystallographica Section C* or *E*, you should make sure that full publication checks are run on the final version of your CIF prior to submission.

Publication of your CIF in other journals

Please refer to the *Notes for Authors* of the relevant journal for any special instructions relating to CIF submission.

PLATON version of 05/02/2014; check.def file version of 05/02/2014

Datablock j115mp-p21 - ellipsoid plot

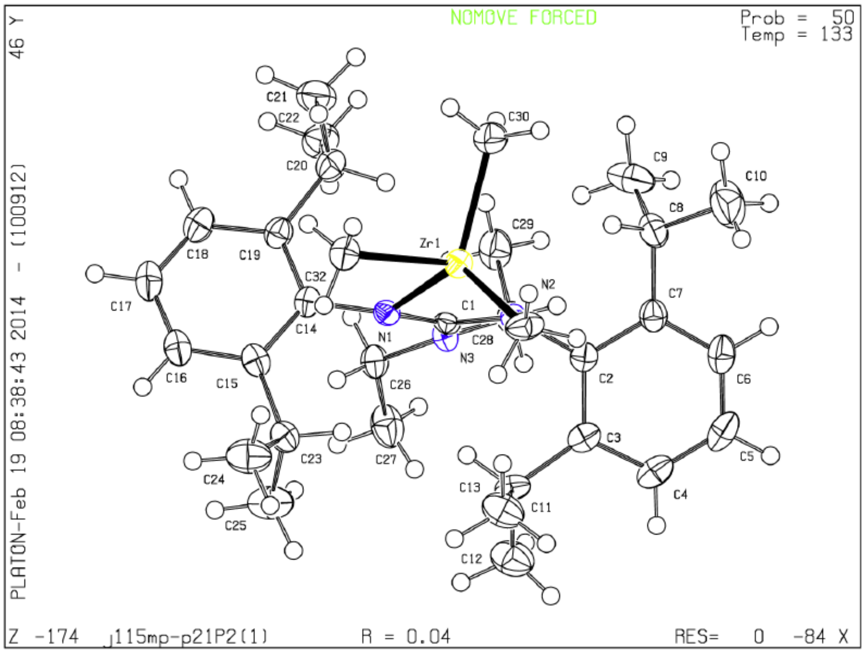

Fig. S 6. Check CIF File compound **I**.

Polymerization

Polymerization of ethylene:

Typical CCTP studies were carried in a batch mode under continuous ethylene flow and constant pressure. The autoclave was heated under vacuum prior to use, cooled to the desired temperature, charged with toluene and saturated with ethylene. CTA (triethyl aluminum), activator and precatalyst (**I**) were applied as stock solutions in toluene and subsequently injected into the autoclave. After the olefin consumption was finished, the autoclave was vented and the reaction was quenched with EtOH. The slurry was cooled with ice. The precipitated polymer was filtrated, washed with EtOH / HCl_{aq} and dried at 50 °C.

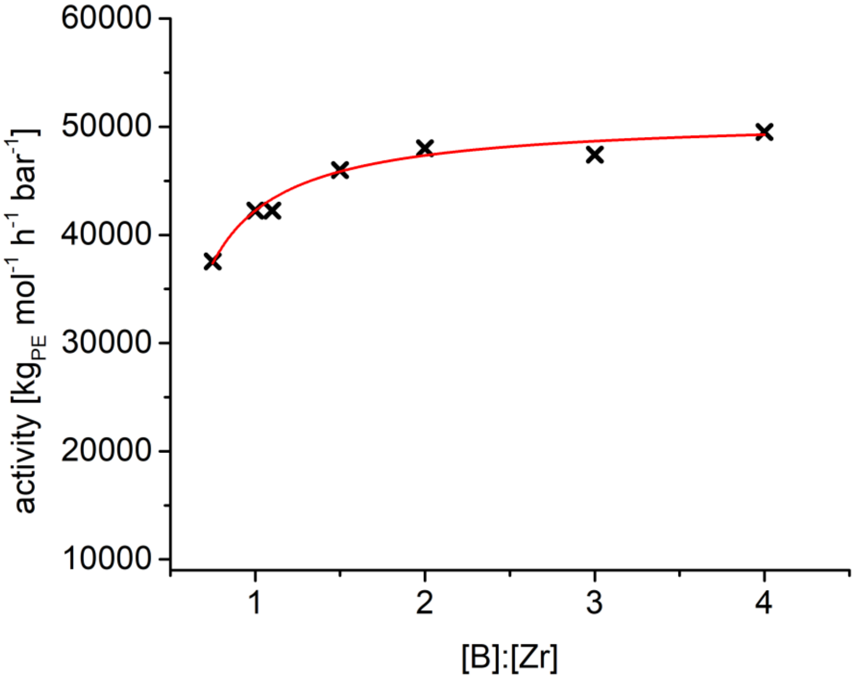

Fig. S 7. Dependency of the polymerization activity by the [B]:[Zr] ratio. B = NH^+B^- Conditions: V_{Tol} = 75 ml; $n_{cat(I)}$ = 0.5 µmol; n_{TEAl} = 2 mmol; $p_{ethylene}$ = 1.5 bara; 1000 rpm, t = 70 °C, v_{Eth} = 3 l.

Table S 4. Dependence of the polymerization activity of **I** in CCTP of ethylene with TEAl at different polymerization temperatures (Figure 3).

Temperature [°C]	Ethylene Consumption[a] [kg_{eth} mol_{cat}^{-1} h^{-1} bar^{-1}]	M_n [g mol^{-1}]	Đ
80	29200	500	1.15
70	32600	650	1.11
60	15900	680	1.14
50	5200	710	1.15

Reaction-conditions: V_{Tol} = 75 ml; $n_{cat(\mathbf{I})}$ = 0.5 µmol; n_{NH+B-} = 0.75 µmol; n_{TEAl} = 3 mmol; $p_{ethylene}$ = 1.5 bara; 1000 rpm. a) Based on the time required until 4 l ethylene were consumed b) N_{exp} = $m_{ethylene}$ / $Mn_{polymer}$-M_{ethyl}; N_{theo} = 3·n(Al).

Table S 5. Dependence of the polymerization activity of **I** in CCTP of ethylene with TEAl at even higher Al:Zr.

Al:Zr	V_{Eth} [l]	Ethylene Consumption[a] [kg_{eth} mol_{cat}^{-1} h^{-1} bar^{-1}]	M_n [g mol^{-1}]	Đ	N_{exp}/N_{thea}[b)] [%]
10000	3	21800	500	1.09	51
20000	6	21200	490	1.10	52
30000	9	14000	460	1.13	54
40000	12	11400	480	1.03	52

Reaction-conditions: T = 70 °C; solvent toluene; $n_{cat(\mathbf{I})}$ = 0.5 µmol; n_{NH+B-} = 0.75 µmol; c_{TEAl} = 66.6 µmol ml^{-1}; $p_{ethylene}$ = 1.5 bara; 1000 rpm. a) Based on the time required until the certain amount of ethylene was consumed b) N_{exp} = $m_{ethylene}$ / $Mn_{polymer}$-M_{ethyl}; N_{theo} = 3·n(Al)

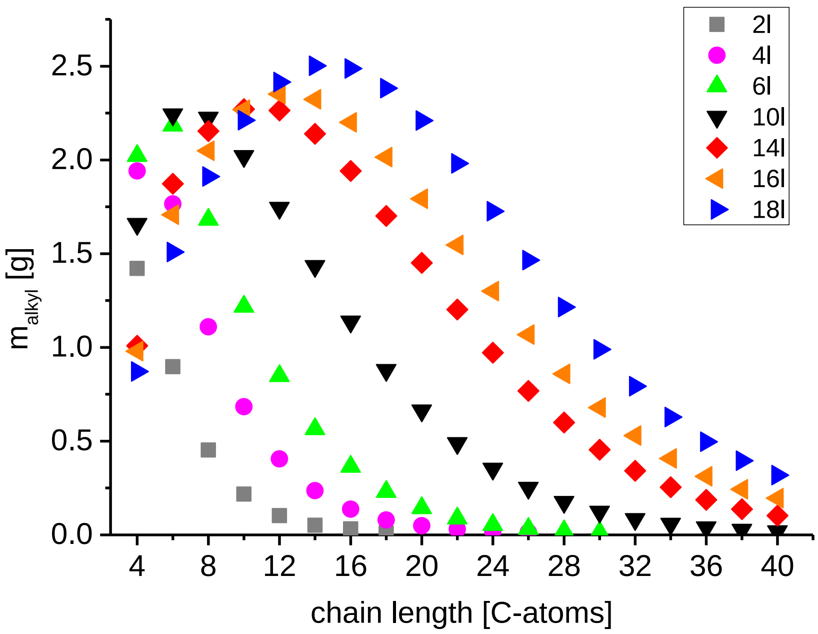

Fig. S 8. Analysis of mass distribution of different alkane fractions synthesized with **I** as precatalyst and variation of the ethylene consumption. (Fig. 5A). Conditions: 1 µmol I; 2 bara ethylene, 250 mL toluene, 50 mmol TEAl, 1.1 µmol $NH^{+}B^{-}$

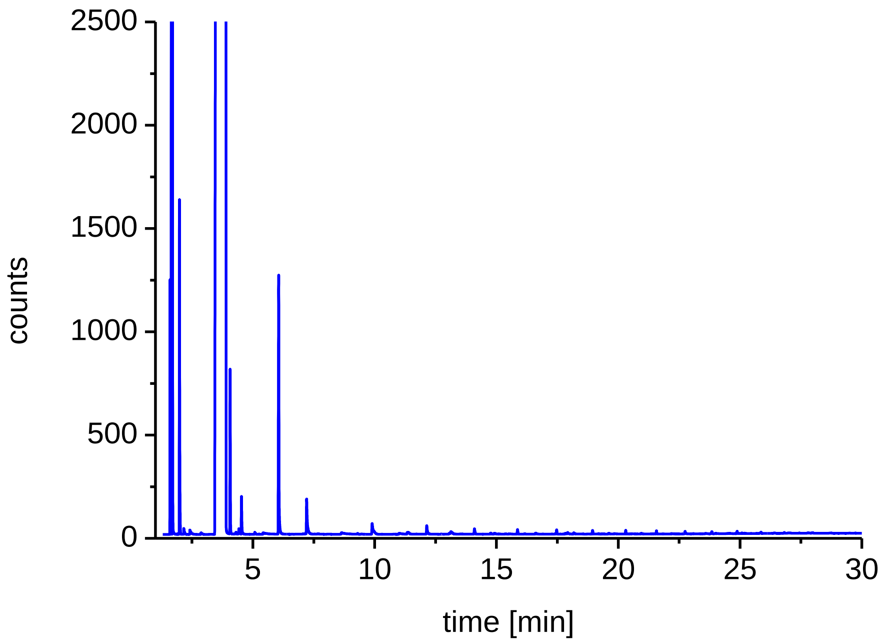

Fig. S 9. GC spectrum of the 2 l sample (**Fig. S 8**).

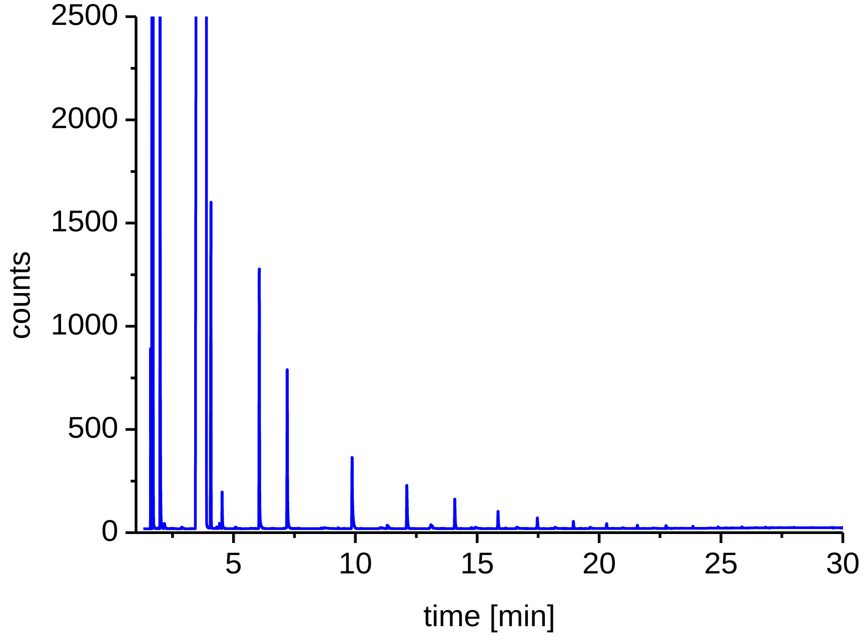

Fig. S 10. GC spectrum of the 4 l sample (**Fig. S 8**).

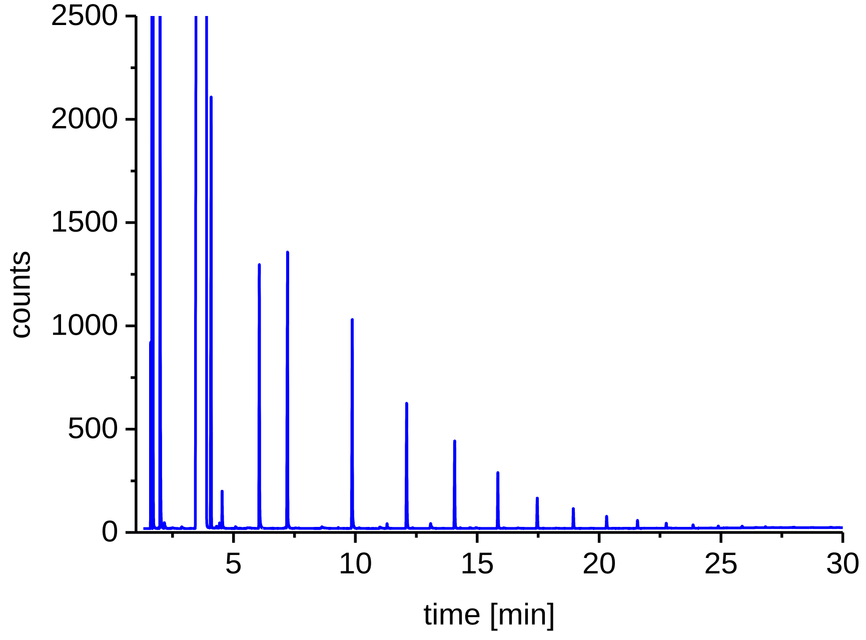

Fig. S 11. GC spectrum of the 6 l sample (**Fig. S 8**).

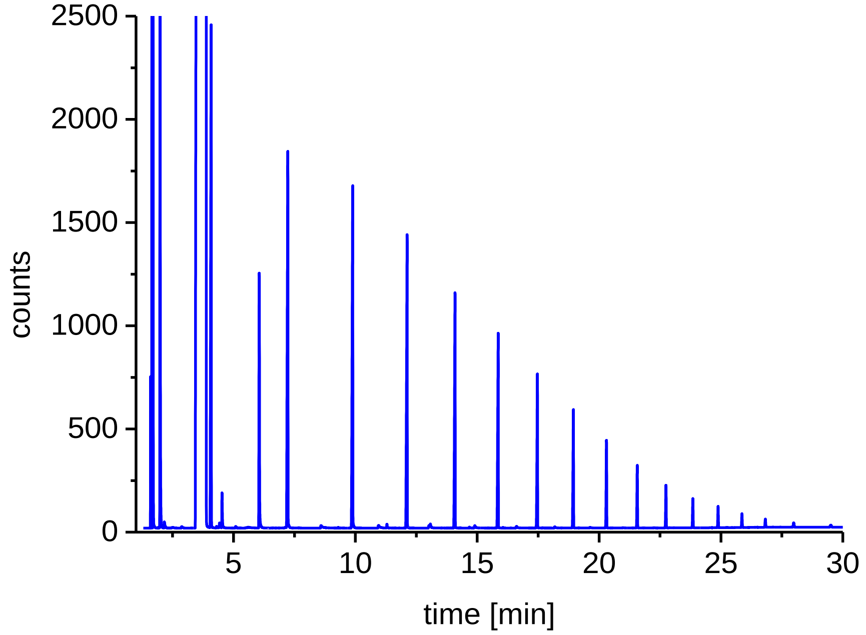

Fig. S 12. GC spectrum of the 10 l sample (**Fig. S 8**).

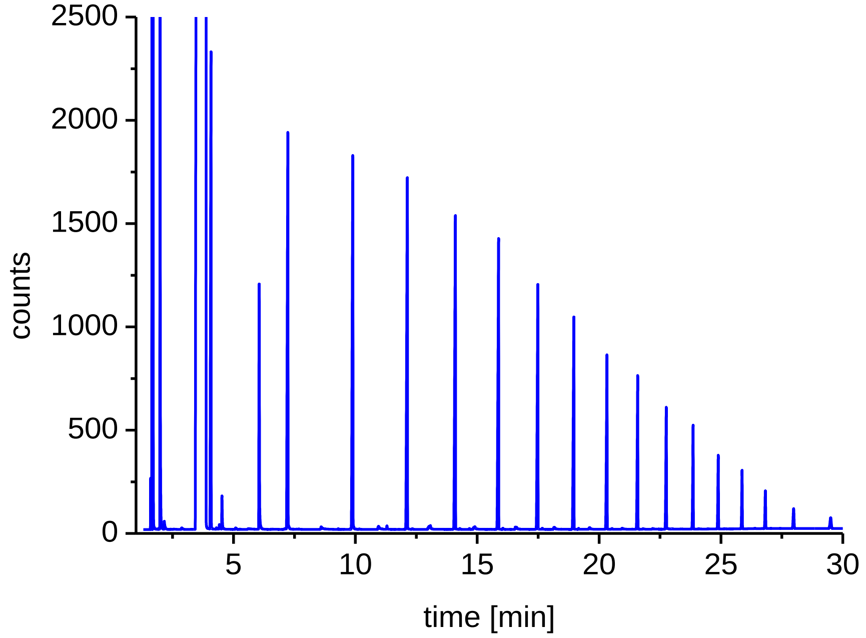

Fig. S 13. GC spectrum of the 14 l sample (**Fig. S 8**).

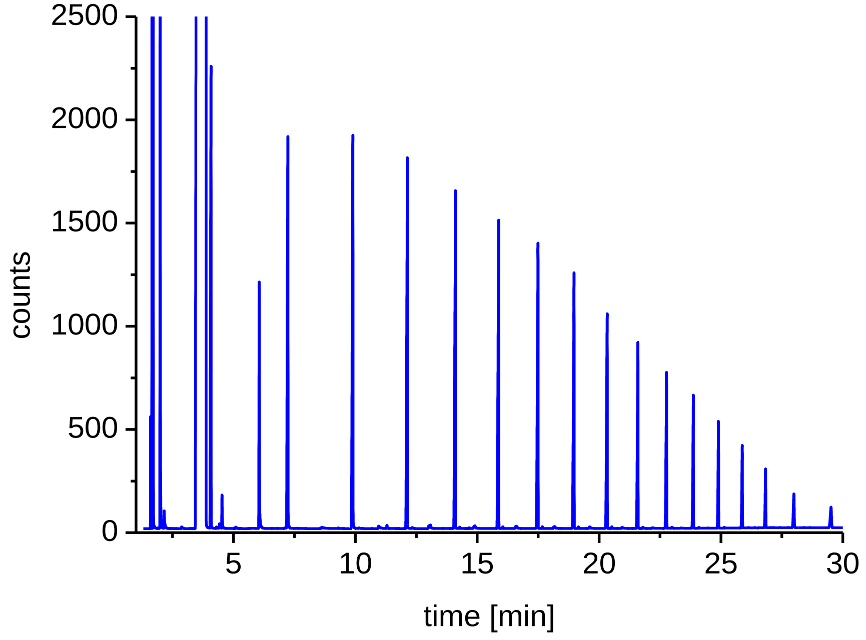

Fig. S 14. GC spectrum of the 16 l sample (**Fig. S 8**).

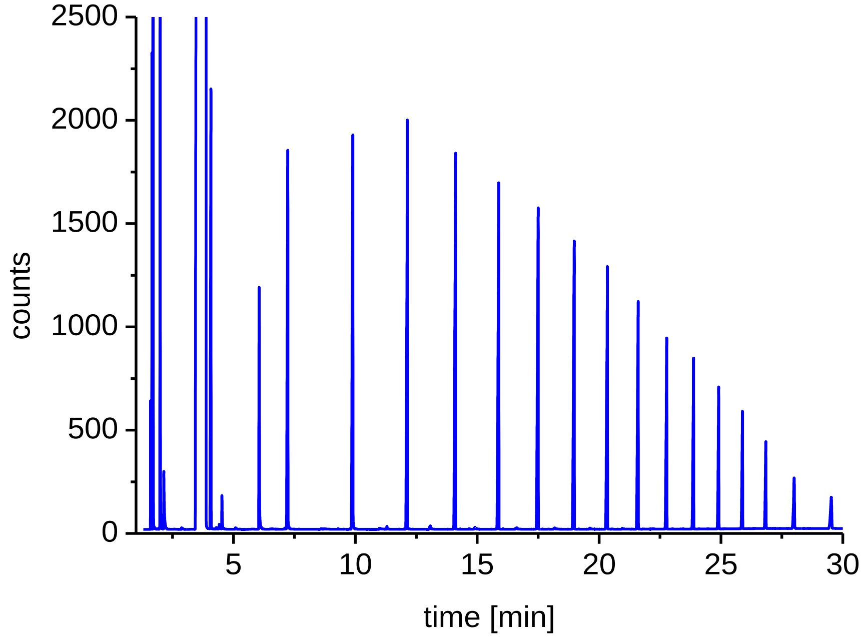

Fig. S 15. GC spectrum of the 18 l sample (**Fig. S 8**).

Table S 6. Analysis of mass distribution of different alkane fractions synthesized with **I** as precatalyst and variation of the ethylene consumption. (Fig. 5A). K is calculated over the whole product spectrum (see Fig. S 16). After 24 l and 98 minutes: 9200 kg $mol^{-1}h^{-1}bar^{-1}$; Mn = 270 g mol^{-1}; Đ = 1.29; total amounts of chains 110000. Conditions: 1 µmol **I**; 2 bara ethylene, 250 mL toluene, 50 mmol TEAl, 1.1 µmol NH^+B^-

m(C24) [g]	m(C26) [g]	m(C28) [g]	m(C30) [g]	m(C32) [g]	m(C34) [g]	m(C36) [g]	m(C38) [g]	m(C40) [g]	K
									0.40
0.02	0.01								0.52
0.05	0.03	0.02	0.01						0.57
0.35	0.25	0.18	0.12	0.08	0.06	0.04	0.03	0.02	0.69
0.97	0.76	0.60	0.45	0.34	0.25	0.19	0.14	0.10	0.78
1.30	1.07	0.86	0.68	0.53	0.41	0.31	0.24	0.20	0.81
1.72	1.46	1.21	0.99	0.79	0.63	0.49	0.39	0.32	0.84

Sample	m(C4) [g]	m(C6) [g]	m(C8) [g]	m(C10) [g]	m(C12) [g]	m(C14) [g]	m(C16) [g]	m(C18) [g]	m(C20) [g]	m(C22) [g]
2l	1.42	0.90	0.45	0.21	0.10	0.05	0.03	0.01		
4l	1.94	1.77	1.11	0.68	0.41	0.24	0.14	0.08	0.05	0.03
6l	2.02	2.18	1.68	1.21	0.85	0.56	0.36	0.23	0.14	0.09
10l	1.66	2.24	2.22	2.02	1.74	1.43	1.14	0.88	0.66	0.49
14l	1.01	1.87	2.15	2.27	2.26	2.14	1.94	1.70	1.45	1.20
16l	0.98	1.71	2.05	2.26	2.35	2.32	2.20	2.01	1.79	1.54
18l	0.87	1.51	1.91	2.21	2.42	2.50	2.48	2.38	2.21	1.98

Experimentally obtained oligomer distributions were analyzed using the Solver add-in for Microsoft Excel which enables a least-square fit of the experimentally obtained molar distributions to the Schulz-Flory formula:

$$C_p = c * (1 - k) * k^{(p-1)}$$

k = k-Value

C_p = amount of oligomer in moles (having p units of monomer inserted)

c = total amount of product in moles

p = number of monomer units inserted in the metal-carbon bond

R^2 = sum of square errors

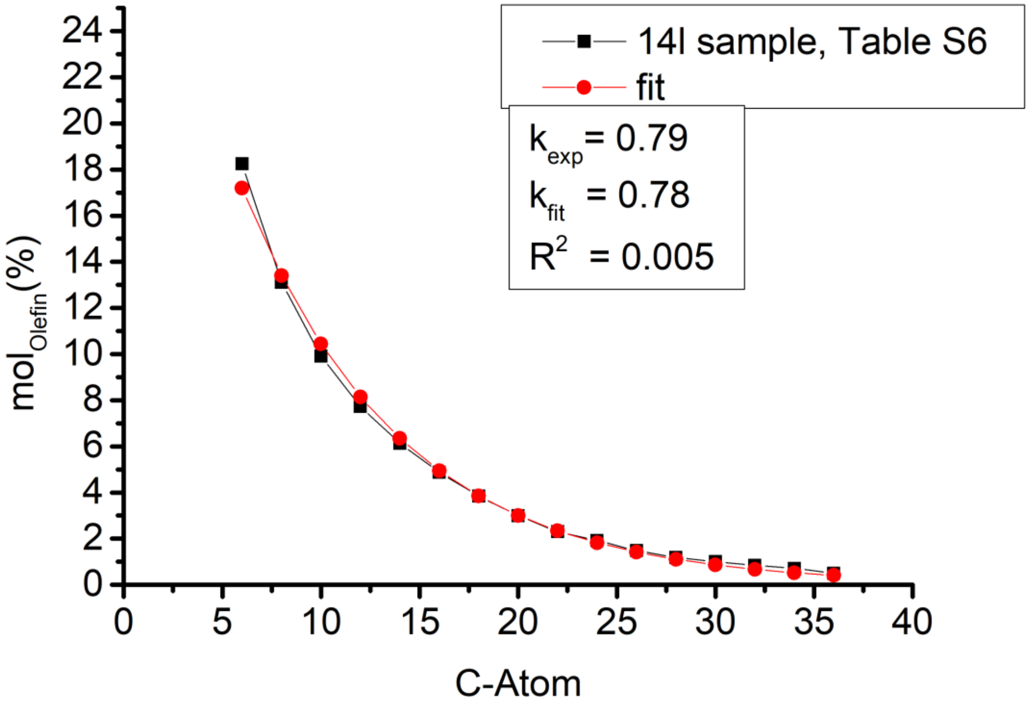

Fig. S 16. Fit and experimental distribution of α-olefins as example

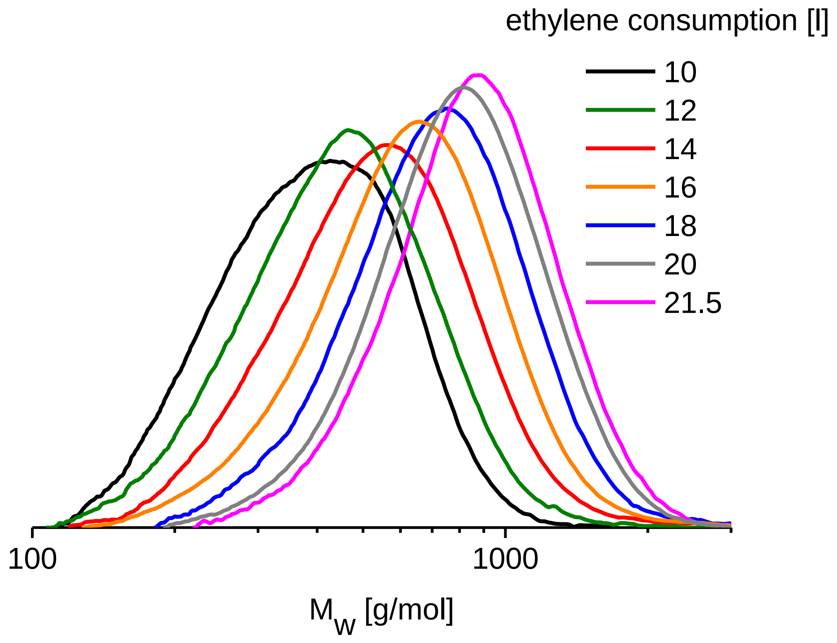

Fig. S 17. SEC graph of HDPE synthesized with **I** as precatalyst and variation of the ethylene consumption. (Fig. 5B)

Table S 7. SEC data of HDPE synthesized with **I** as precatalyst and variation of the ethylene consumptions.

**entry**	**V_{eth} [l]**	**$Đ$**	**M_n [g mol^{-1}]**	**N_{exp}/N_{theo} a) [%]**
1	10	1.2	350	89
2	12	1.2	400	95
3	14	1.2	470	96
4	16	1.2	550	96
5	18	1.2	650	95
6	20	1.2	720	97
7	21.5	1.2	780	99

Reaction-conditions: T = 70 °C; V_{Tol} = 275 ml; $n_{cat(\mathbf{I})}$ = 1.0 µmol; n_{NH+B-} = 1.5 µmol; n_{TEAl} = 14.6 mmol; $p_{ethylene}$ = 1.5 bara; 1000 rpm. a) N_{exp} = $m_{ethylene}$ / $Mn_{polymer}$-M_{ethyl}; N_{theo} = 3·n(Al). The GPC measurement for the 10 and 12 l experiments were taken directly from toluene solution. The others were measured from dried polymer solids.

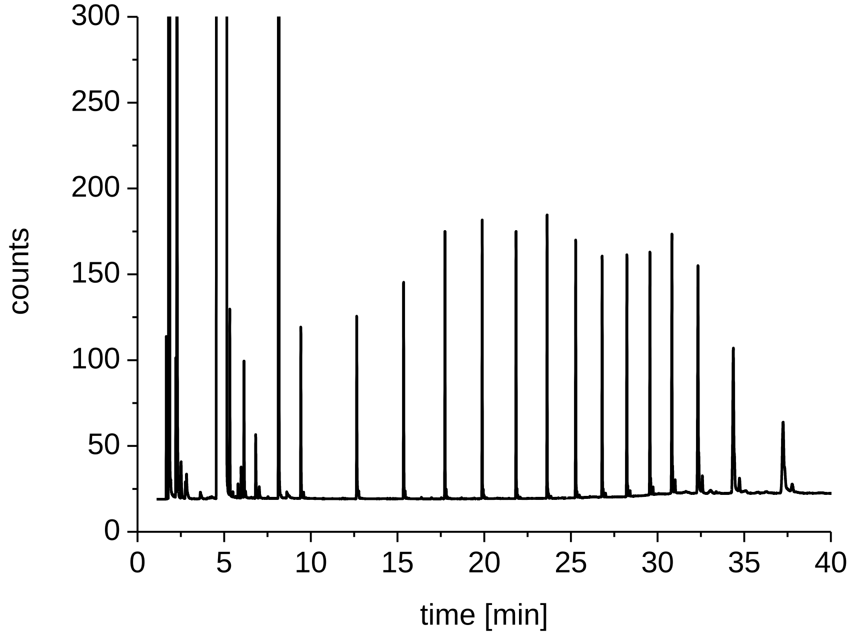

Fig. S 18. GC graph of HDPE synthesized with **I** (**Table S 7**, entry 1) as precatalyst and extracted with ethanol while work up.

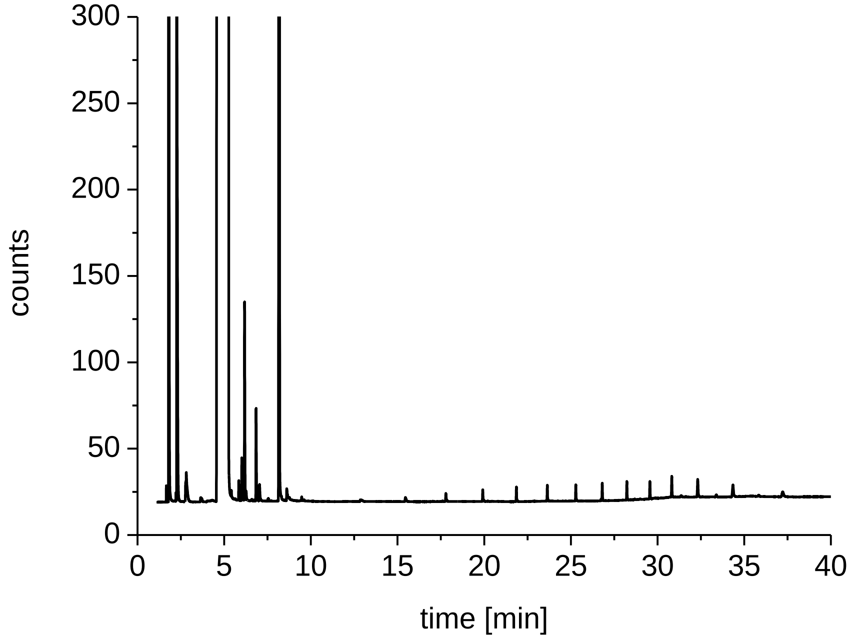

Fig. S 19. GC graph of HDPE synthesized with **I** (**Table S 7**, entry 4) as precatalyst and extracted with ethanol while work up.

Synthesis of HDPE-OH

Instead of quenching with EtOH the autoclave was vented and pressurized with 2 bar of dry oxygen and heated up to 80°C for 2 hours. Afterwards the slurry was cooled to room temperature and treated as described above.

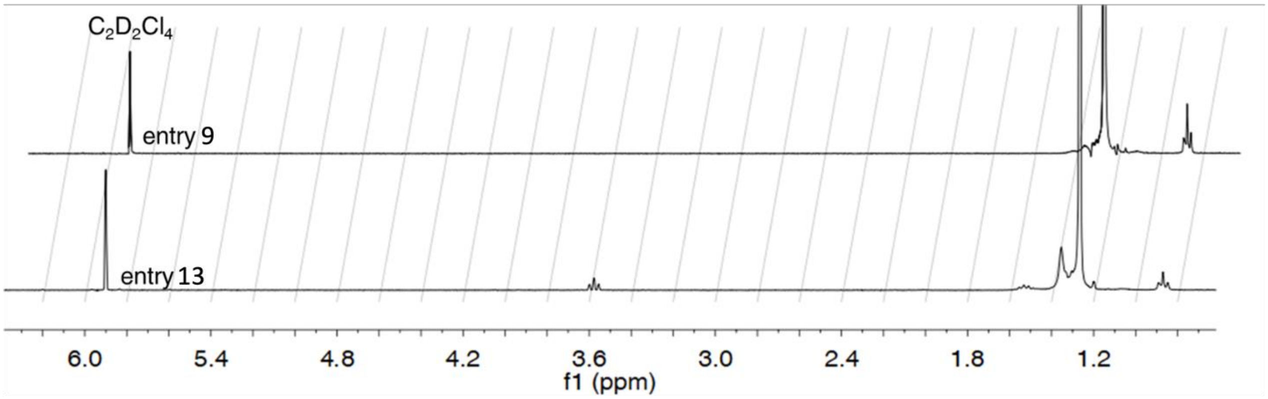

Fig. S 20. ^{1}H-NMR spectrum of the products measured in $C_2D_2Cl_4$ at 120 °C of entry 9 and 13 of table 2. The triplet signal at 3.6 ppm (CH_2-OH) indicates the oxidation of the metal terminated chain-end in the reaction yielding a polymer alcohol (69 mol%) after acidic workup.

Table S 8. Dependence of the polymerization activity of **I** by the inverse Al concentration (Fig. 6C).

1/c Al [ml mmol^{-1}]	Ethylene Consumption[a] [kg_{eth} mol_{cat}^{-1} h^{-1} bar^{-1}]	M_n [g mol^{-1}]	Đ	N_{exp}/N_{thea}[b)] [%]
50	54600	950	1.17	88
38	45500	760	1.18	83
33	41700	650	1.15	78
25	32600	650	1.11	65
19	26700	520	1.11	60
15	21800	500	1.09	51
10	14600		liquid	

Reaction-conditions: T = 70 °C; V_{Tol} = 75 ml; $n_{cat(\mathbf{I})}$ = 0.5 µmol; n_{NH+B-} = 0.75 µmol; $V_{ethylene}$ = 3 l; $p_{ethylene}$ = 1.5 bara; 1000 rpm. a) Based on the time required until 3 l ethylene were consumed b) $N_{exp} = m_{ethylene} / Mn_{polymer}\text{-}M_{ethyl}$; $N_{theo} = 3 \cdot n(Al)$.

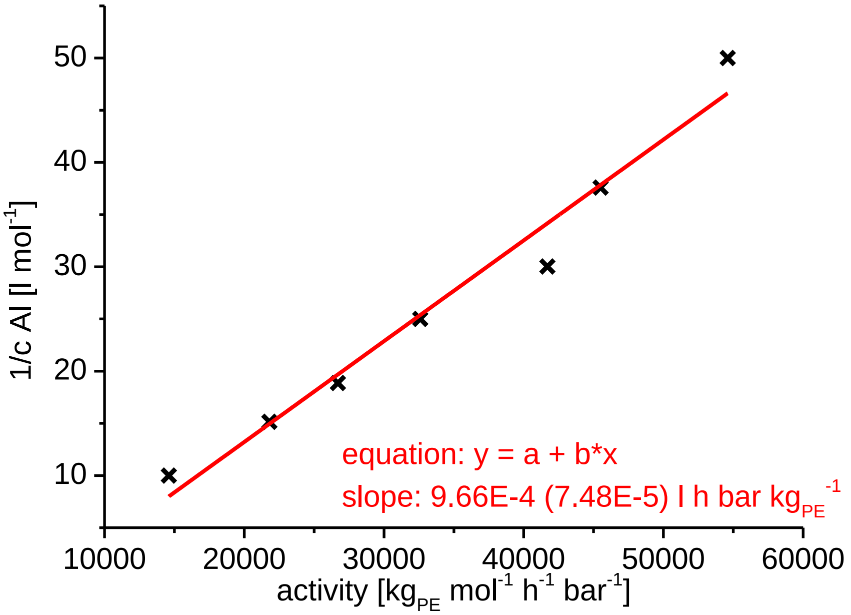

Fig. S 21. Dependency of the polymerization activity by the inverse Al concentration. Conditions: 1.5 bar ethylene, 3 l ethylene, 0.5 µmol **I**, 0.75 µmol NH^+B^-, 75 ml toluene.

*Mechanistical consideration of the activation process of **I** and the polymerization:*

17.1 mg of **I** was dissolvend in 0.6 ml C_6D_5Br in a J-Young NMR tube and characterized via ^{1}H-NMR at -30 °C. Then 1 equivalent of NH^+B^- (24.0 mg) was added at -30 °C and characterized via ^{1}H- and ^{19}F-NMR.

^{1}H NMR (400 MHz, C_6D_5Br, -30 °C): δ = 7.25–7.34 [m, 6 H, Ar*H*]; 6.94 [t, ^3J = 7.2 Hz, 2 H, *m*-CAr*H*]; 6.34 [d, ^3J = 8.4 Hz, 2 H, *o*-CAr*H*]; 6.30 [t, ^3J = 7.2 Hz, 1 H, *p*-CAr*H*]; 3.23 [m, 4 H, C*H*(CH_3)$_2$]; 2.86 [q, ^3J = 6.8 Hz, 4 H, N(C$H_2$$CH_3$)$_2$]; 2.73 [s, 6 H, N(C$H_3$)$_2$]; 1.33 [d, ^3J = 2.8 Hz, 12 H, CH(CH_3)$_2$]; 1.21 [d, ^3J = 2.4 Hz, 12 H, CH(CH_3)$_2$]; 0.38 [t, ^3J = 7.2 Hz, 6 H, N(CH_2CH_3)$_2$]; 0.37 [s, 6 H, ZrCH_3] ppm.

14.6 mg of **I** + 1 equivalent TMAl were dissolved in CD_2Cl_2 in a J-Young NMR tube and characterized via ^{1}H-NMR (Fig. S 34).

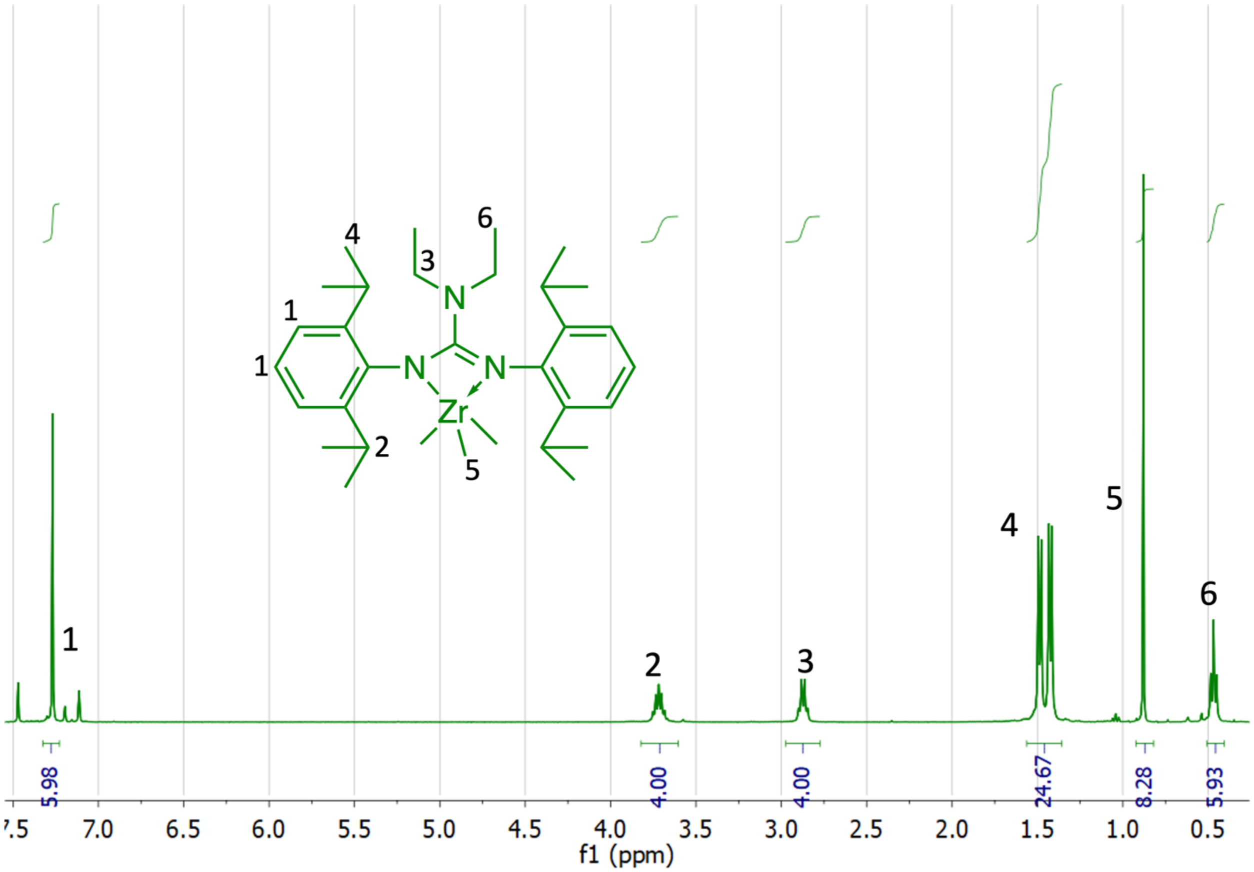

Fig. S 22. 400 MHz ^{1}H NMR study of **I** at -30 °C in C_6D_5Br.

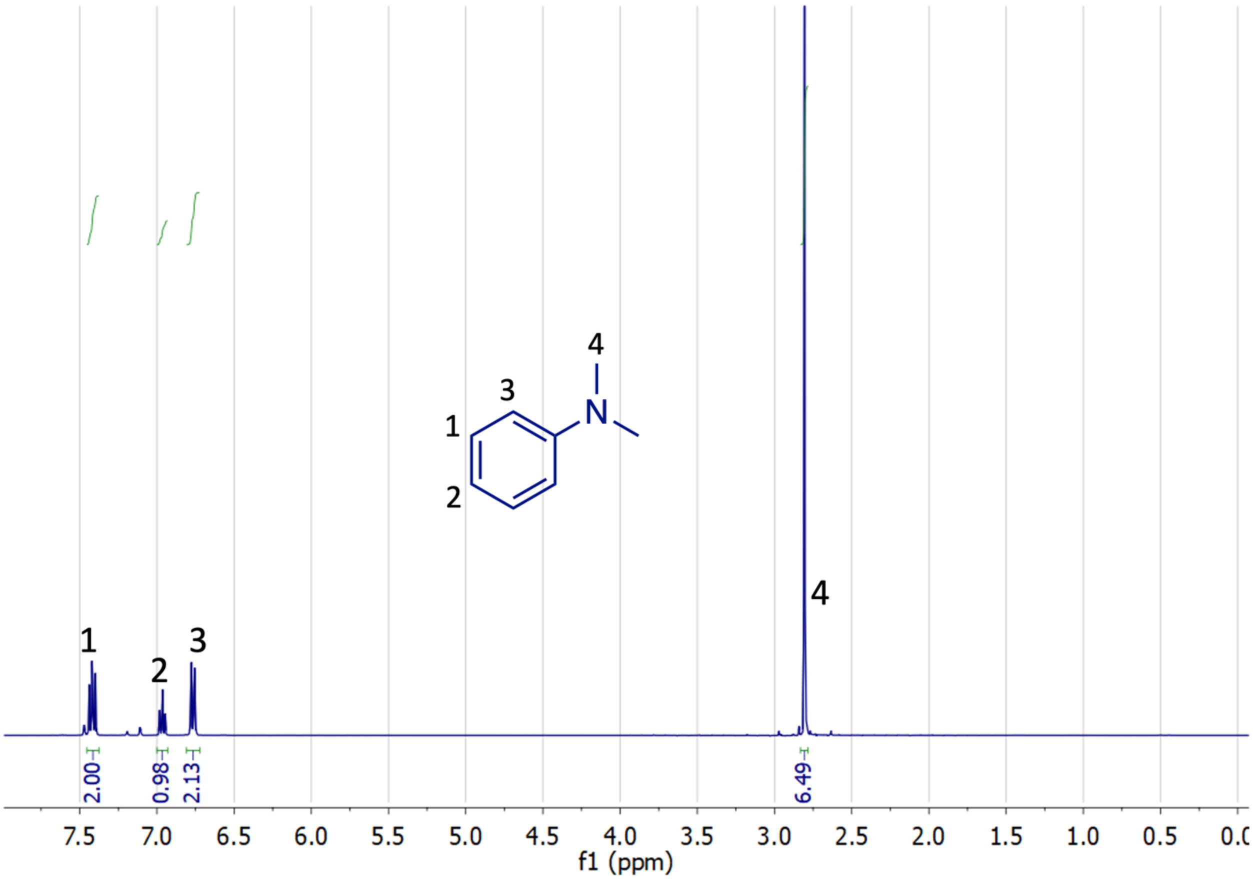

Fig. S 23. 400 MHz ^{1}H NMR study of N,N-dimethylaniline at -30 °C in C_6D_5Br.

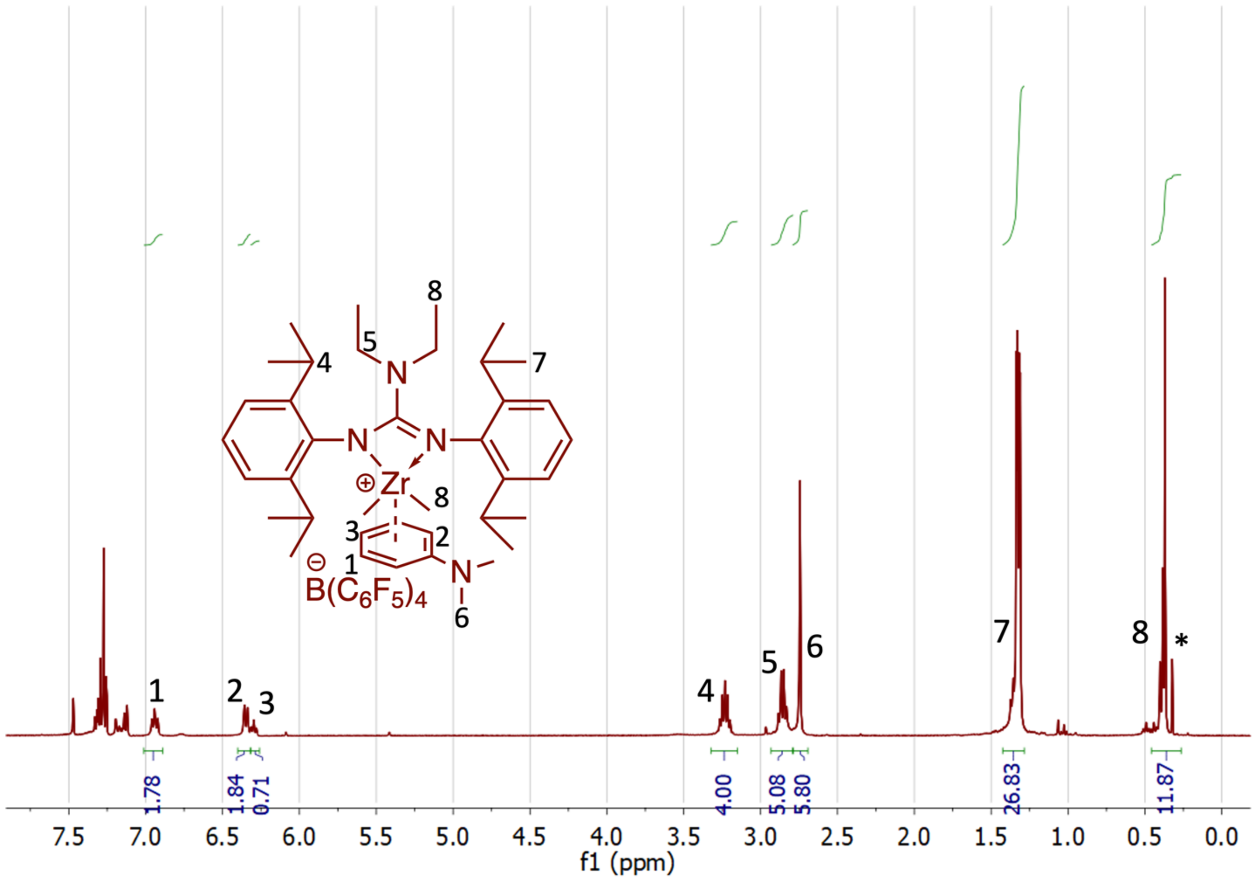

Fig. S 24. 400 MHz ^{1}H NMR study of **I** + 1 equivalent of NH^+B^- at -30 °C in C_6D_5Br.

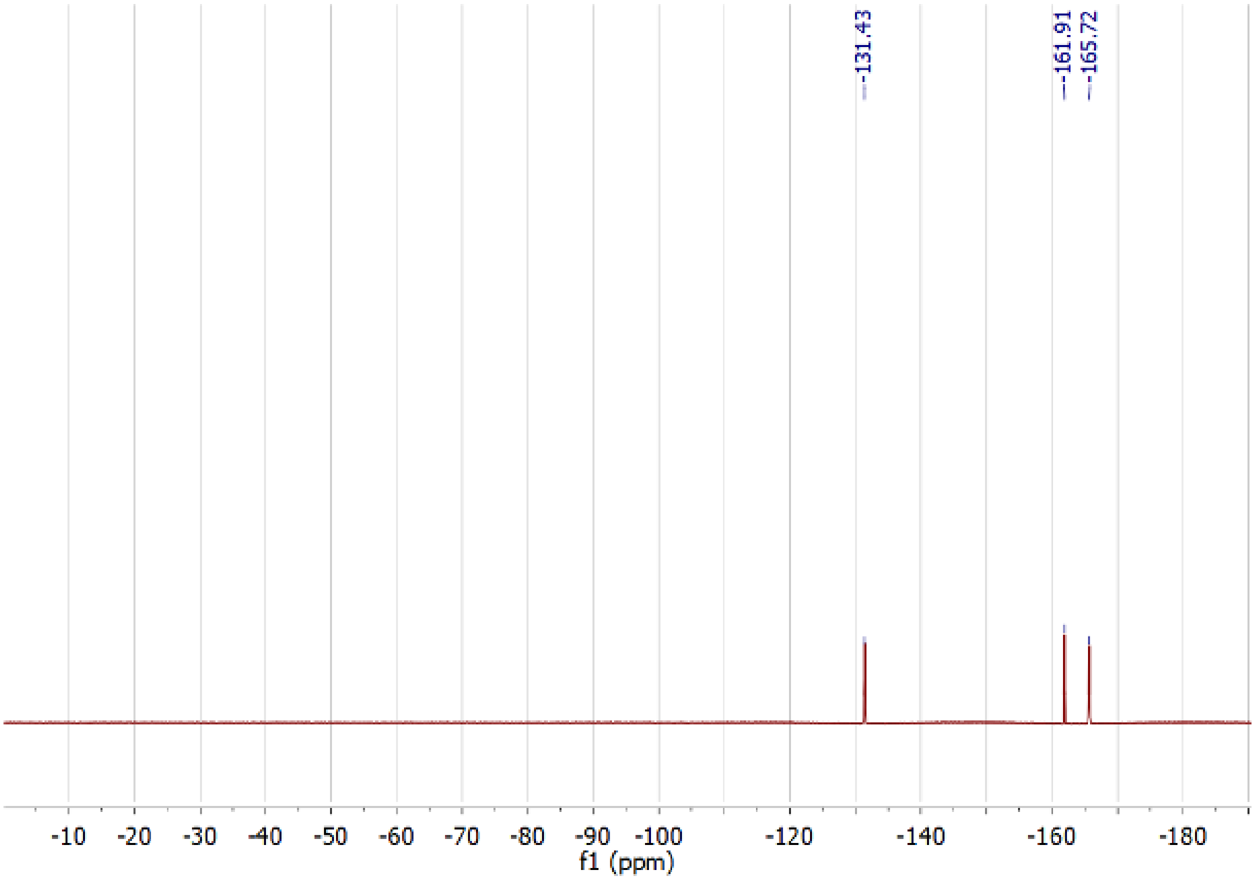

Fig. S 25. 376 MHz ^{19}F NMR study of **I** + 1 equivalent of NH^+B^- at -30 °C in C_6D_5Br.

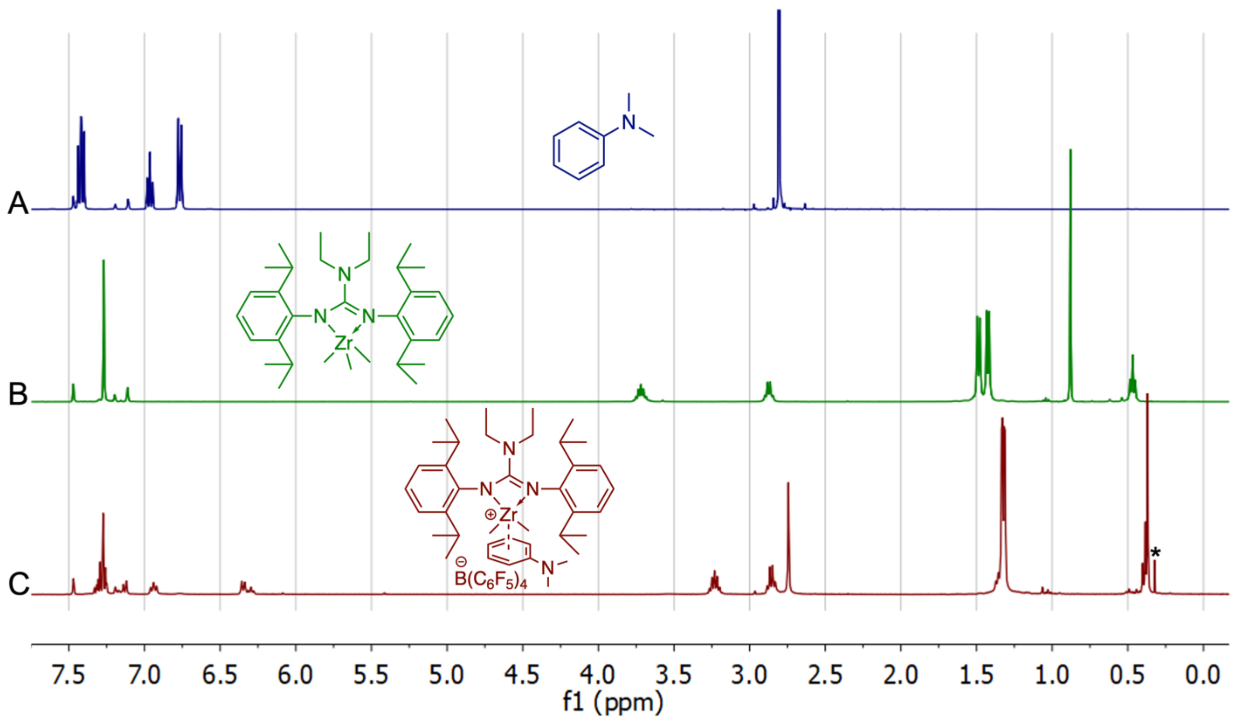

Fig. S 26. 400 MHz ^{1}H NMR study of A) N,N-dimethylaniline; B) **I**; C) **I** + 1 equivalent of $NH^{+}B^{-}$ at -30 °C in C_6D_5Br.

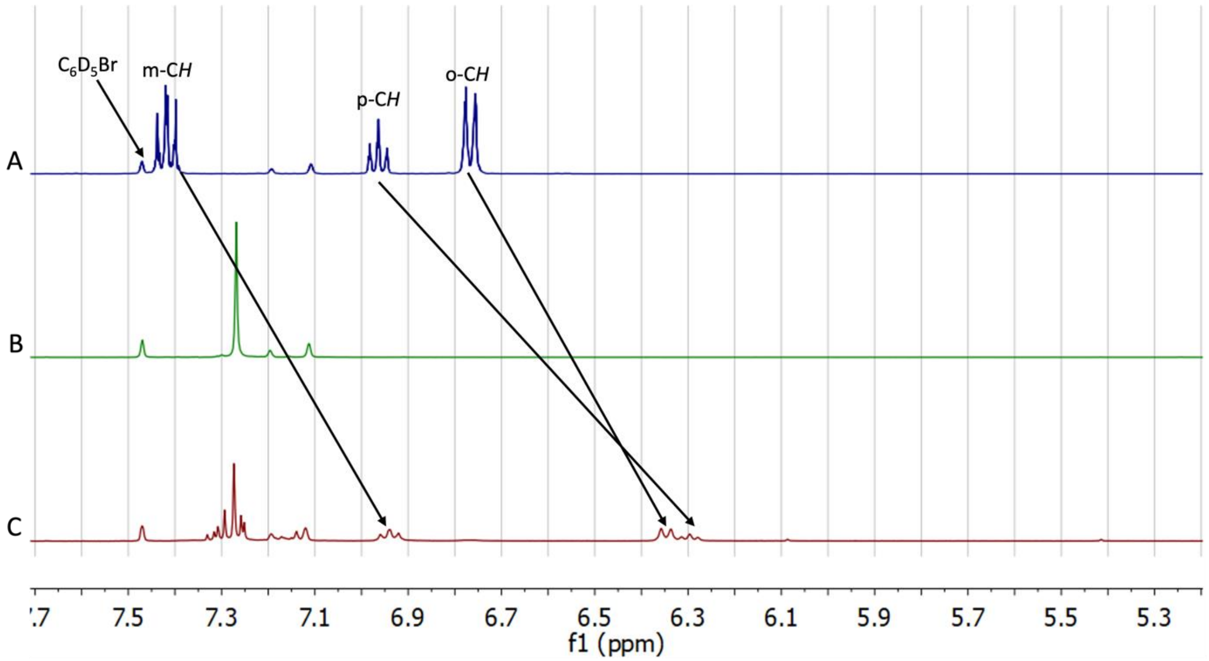

Fig. S 27. 400 MHz ^{1}H NMR study of A) N,N-dimethylaniline; B) **I**; C) **I** + 1 equivalent of $NH^{+}B^{-}$ at -30 °C in C_6D_5Br low-field zoom. The shifting resonances due to coordination are indicated by the arrows.

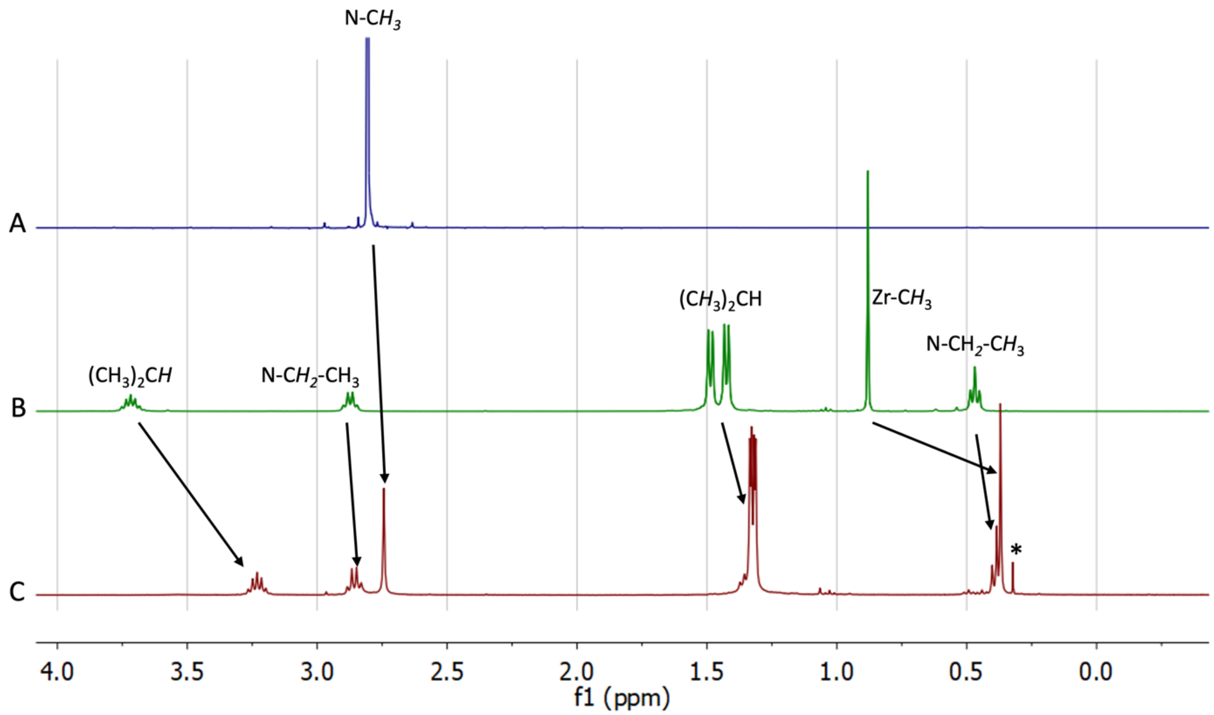

Fig. S 28. 400 MHz ^{1}H NMR study of A) N,N-dimethylaniline; B) **I**; C) **I** + 1 equivalent of $NH^{+}B^{-}$ at -30 °C in C_6D_5Br high-field zoom. The shifting resonances due to coordination are indicated by the arrows. * shows the formation of methane.

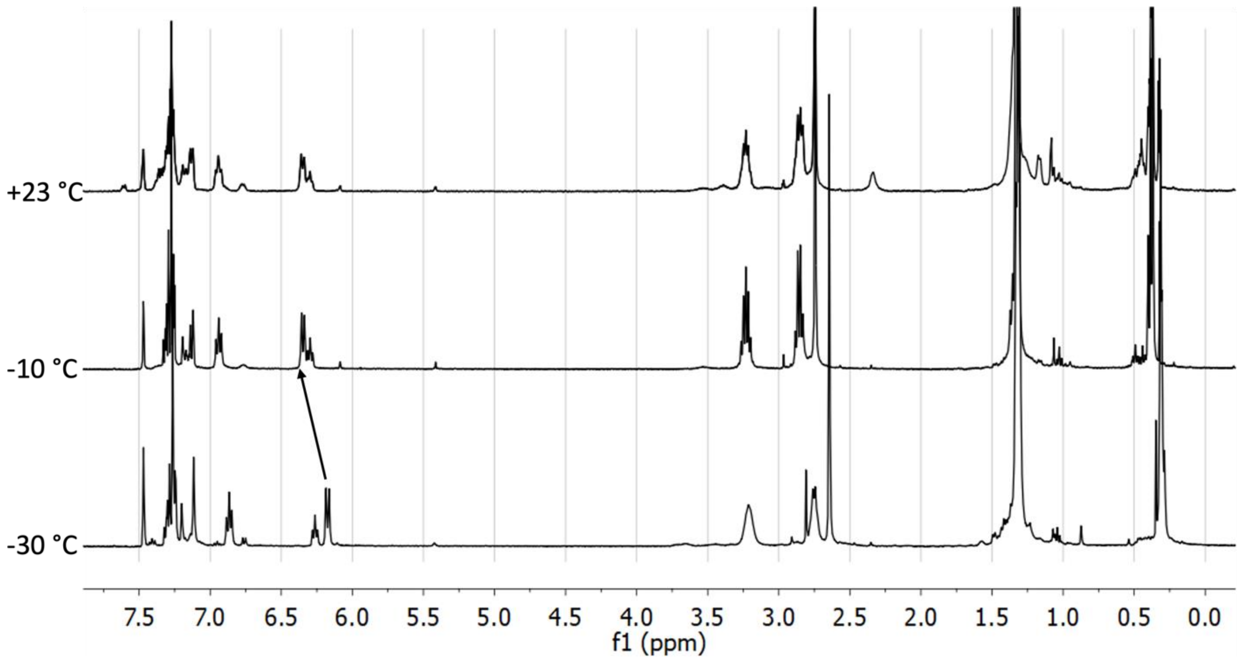

Fig. S 29. Temperature dependent 400 MHz ^1H NMR study of **I** + 1 equivalent of NH^+B^- in C_6D_5Br. The doublet signal of 6.17 ppm is low-field shifted towards 6.34 ppm when the sample is heated up. This is indicated with the arrow.

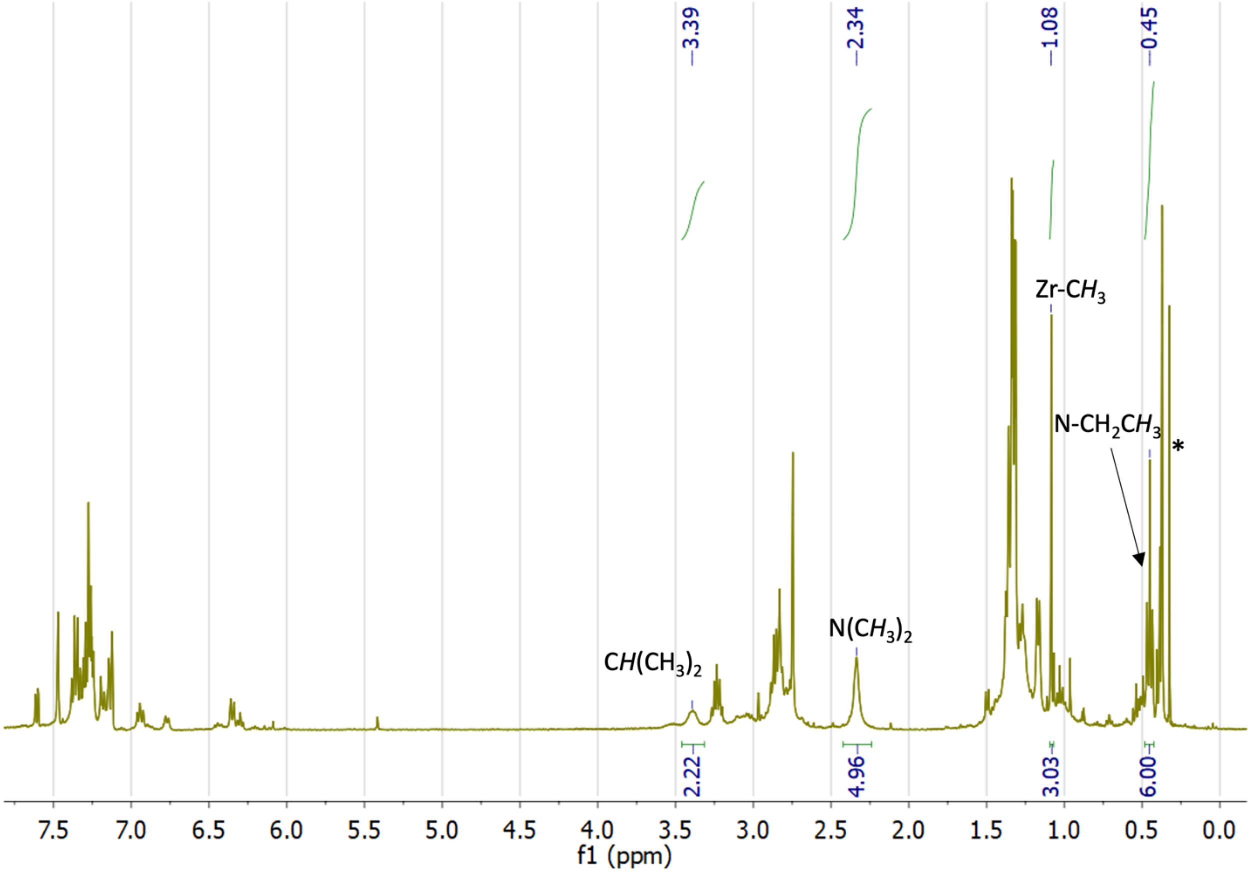

Fig. S 30. 400 MHz ^{1}H NMR study of **I** + 1 equivalent of $NH^{+}B^{-}$ after 4 days and CH-activation of ligand in C_6D_5Br. The new proton resonances are indicated in the spectra. * shows the formation of methane.

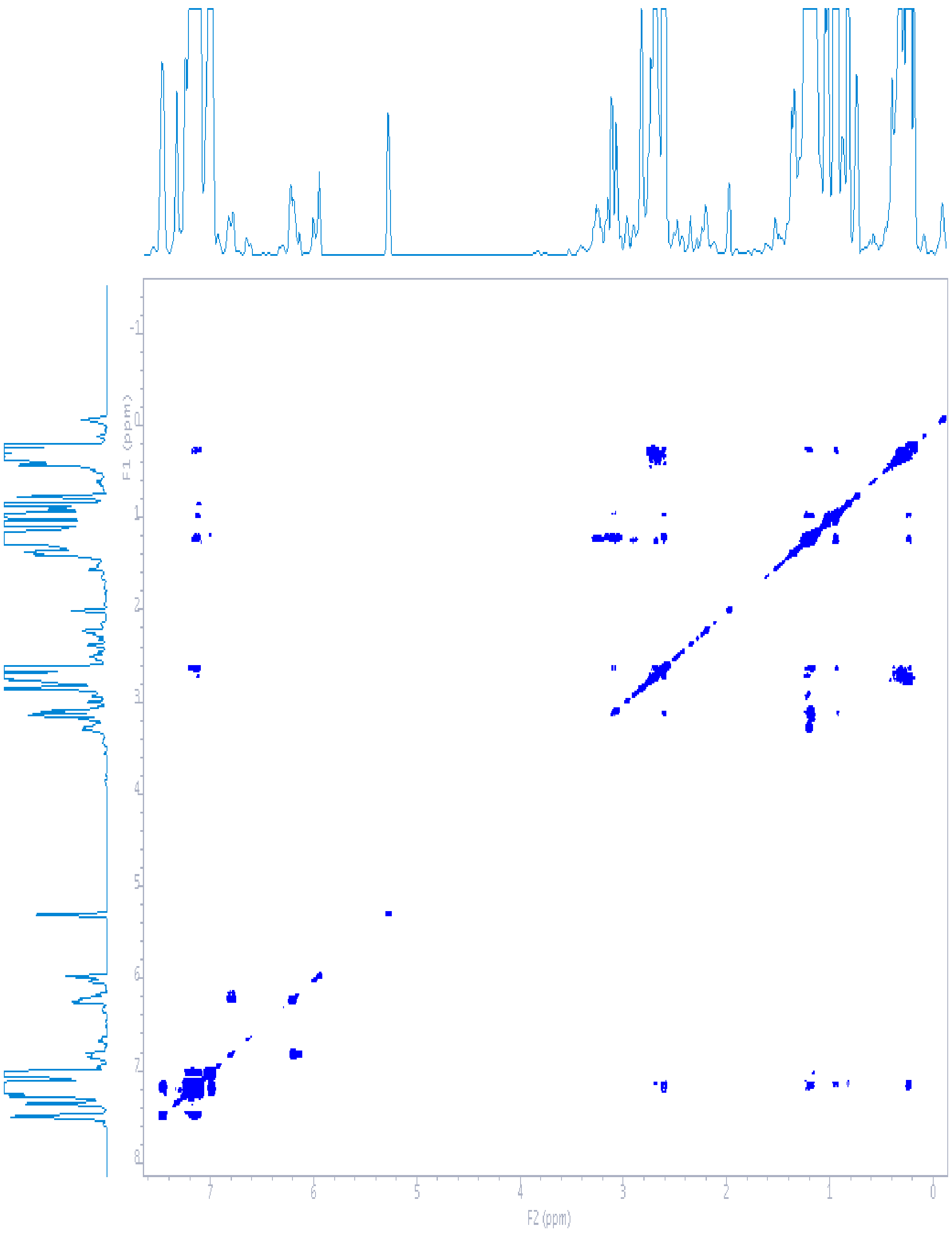

Fig. S 31. COSY of **I** + 1 equivalent of NH^+B^- after 4 days and CH-activation of ligand in C_6D_5Br. (overview)

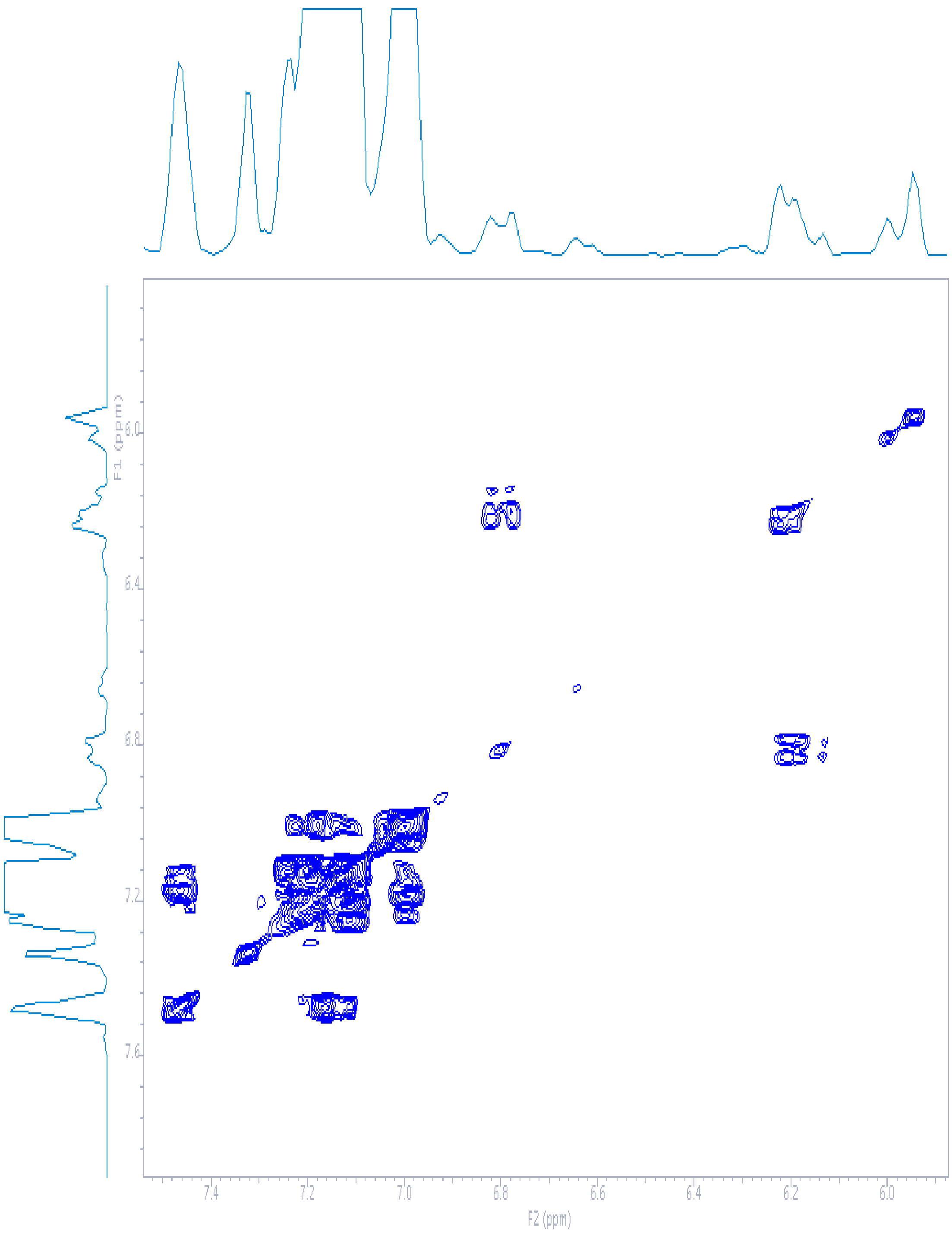

Fig. S 32. COSY of **I** + 1 equivalent of NH^+B^- after 4 days and CH-activation of ligand in C_6D_5Br. (low-field zoom)

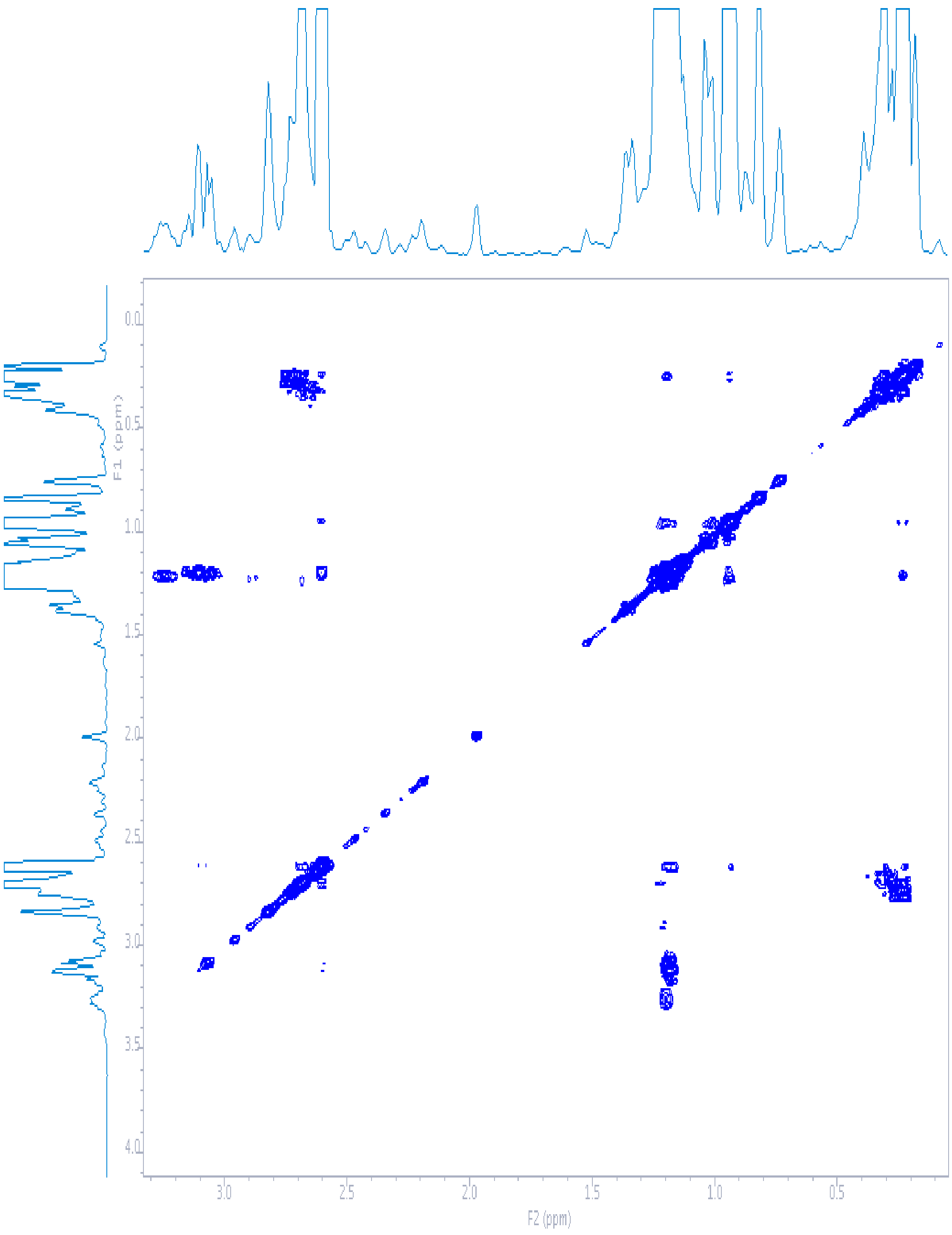

Fig. S 33. COSY of **I** + 1 equivalent of NH^+B^- after 4 days and CH-activation of ligand in C_6D_5Br. (high-field zoom)

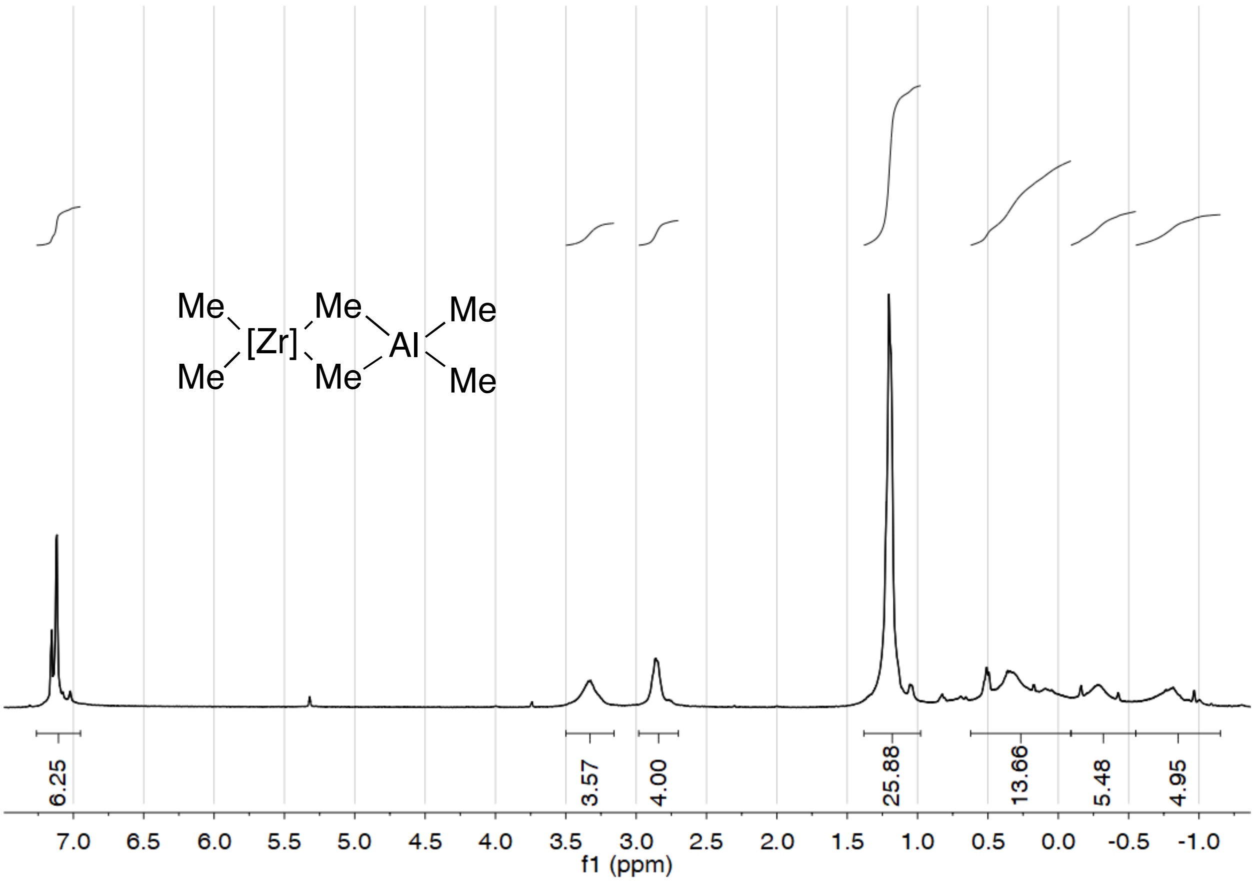

Fig. S 34. 400 MHz ^{1}H NMR study of **I** + 1 equivalent of TMAl at 5 °C in CD_2Cl_2.

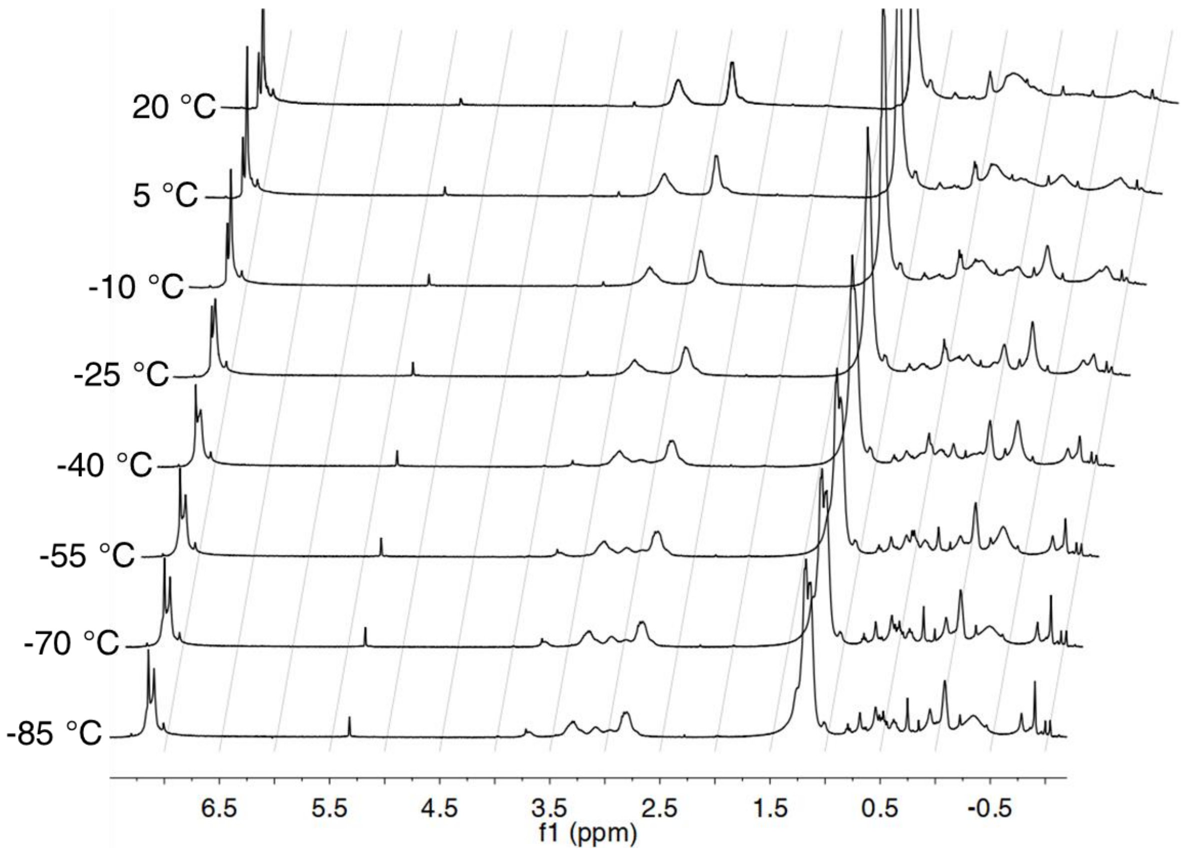

Fig. S 35. Temperature dependent 400 MHz ^{1}H NMR study of **I** + 1 equivalent of TMAl in CD_2Cl_2.

References

[1] a) D. C. Bradley, I. M. Thomas, *J. Chem. Soc.* **1960**, 3857-3861; b) G. M. Diamond, R. F. Jordan, J. L. Petersen, *Organometallics* **1996**, *15*, 4030-4037.

[2] E. V. Avtomonov, K. A. Rufanov, *Z. Naturforsch., B: Chem. Sci.* **1999**, *54*, 1563-1567.

[3] A. Altomare, M. C. Burla, M. Camalli, G. L. Cascarano, C. Giacovazzo, A. Guagliardi, A. G. G. Moliterni, G. Polidori, R. Spagna, *J. Appl. Crystallogr.* **1999**, *32*, 115-119.

[4] G. M. Sheldrick, *Acta Crystallogr., Sect. A: Found. Crystallogr.* **2008**, *64*, 112-122.

[5] L. J. Farrugia, *J. Appl. Crystallogr.* **2012**, *45*, 849-854.

[6] J. S. Rogers, G. C. Bazan, *Chem Comm* **2000**, 1209-1210.

[7] G. Mani, F. P. Gabbai, *Angew. Chem. Int. Ed. Engl.* **2004**, *43*, 2263-2266.
